# Supplementary material for: A deep-learning-based framework for identifying and localizing multiple abnormalities and assessing cardiomegaly in chest X-ray
Source: Nat Commun. 2024 Feb 14;15:1347. doi: 10.1038/s41467-024-45599-z (PMC10867134; doi:10.1038/s41467-024-45599-z)
Supplement: Supplementary file 1 — Supplementary Information [file 41467_2024_45599_MOESM1_ESM.pdf]

A deep-learning-based framework for identifying and localizing multiple abnormalities and assessing cardiomegaly in Chest X-ray

Supplementary Materials

Supplementary Figures and Figure Legends

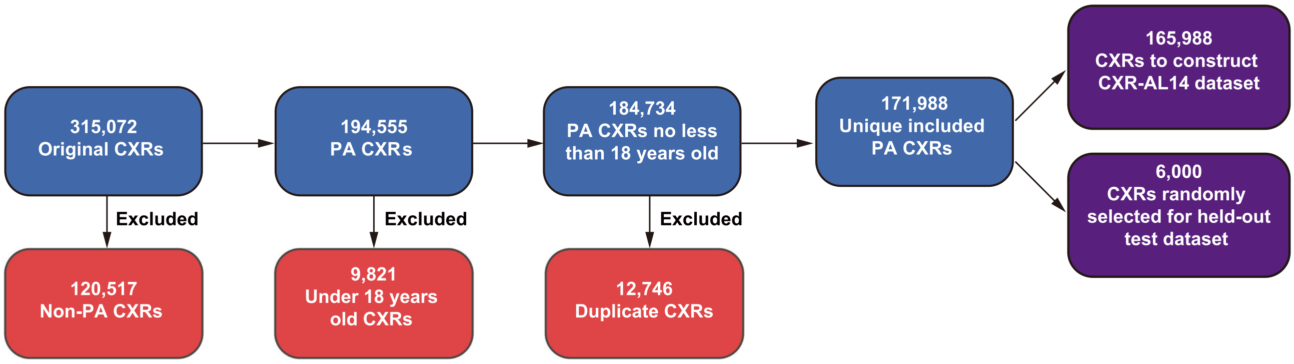

Supplementary Figure 1 Screening process used for the CXRs in this study.
PA: posteroanterior.

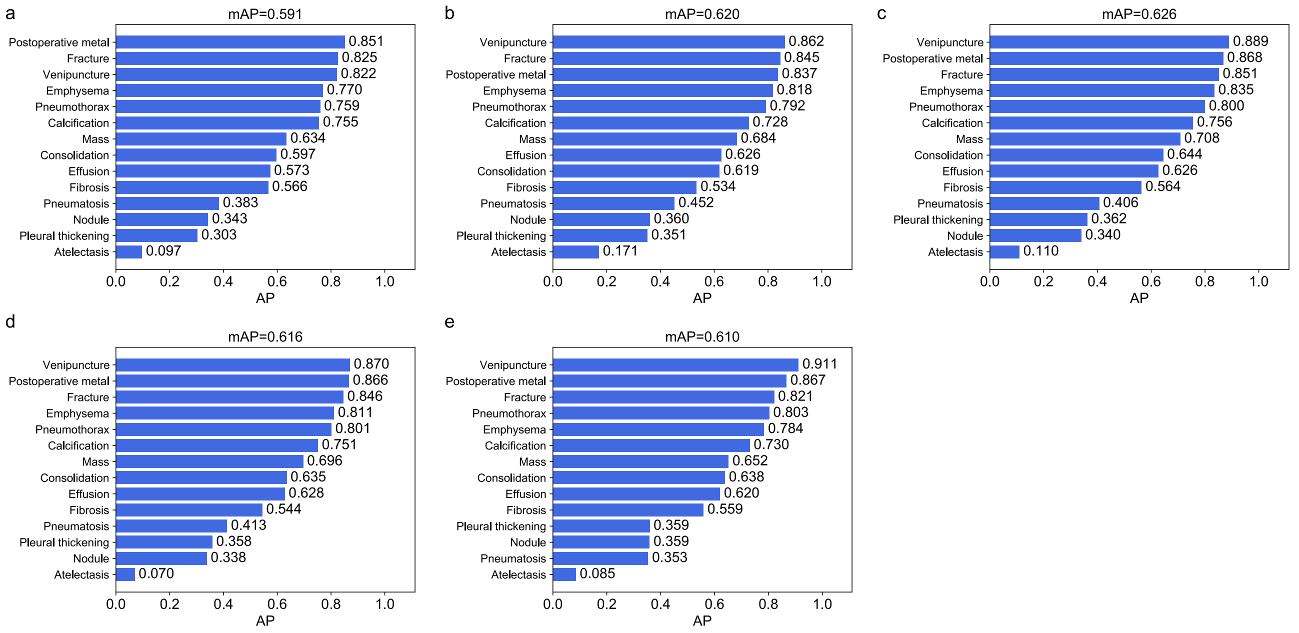

Supplementary Figure 2 Performance of the five models generated by 5-fold cross-validation with an IoU-T of 0.5

a The result of the first fold cross-validation; **b** The result of the second fold cross-validation; **c** The result of the third fold cross-validation; **d** The result of the fourth fold cross-validation; **e** The result of the fifth fold cross-validation. mAP: mean average precision, IoU-T: intersection over union threshold.

(Source data are provided as a Source Data file.)

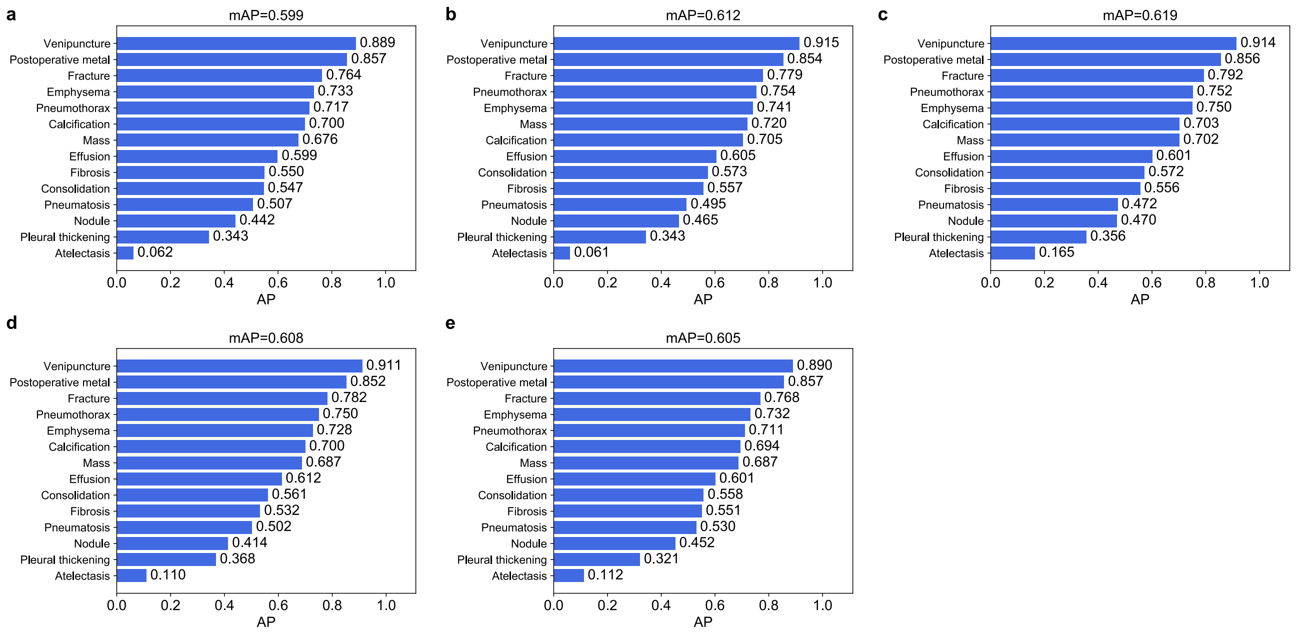

Supplementary Figure 3 Performance of the five models generated by 5-fold cross-validation on the held-out test dataset with an IoU-T of 0.5

a The result of the first fold cross-validation; **b** The result of the second fold cross-validation; **c** The result of the third fold cross-validation; **d** The result of the fourth fold cross-validation; **e** The result of the fifth fold cross-validation. mAP: mean average precision, IoU-T: intersection over union threshold.

(Source data are provided as a Source Data file.)

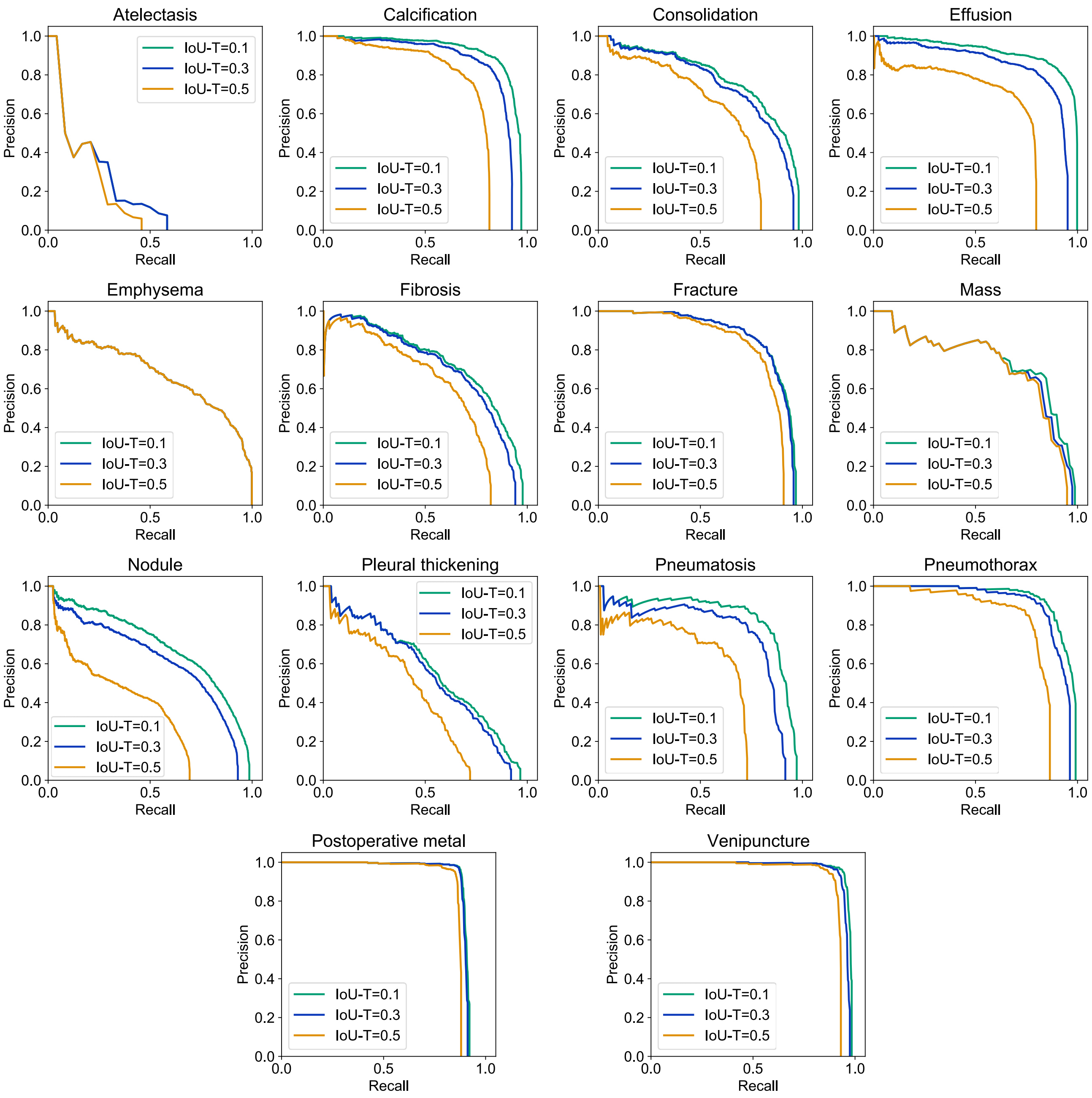

Supplementary Figure 4 PR curves obtained for each chest abnormality achieved by the YOLOX model on the held-out test dataset with different IoU-Ts.

IoU-T: intersection over union threshold.

(Source data are provided as a Source Data file.)

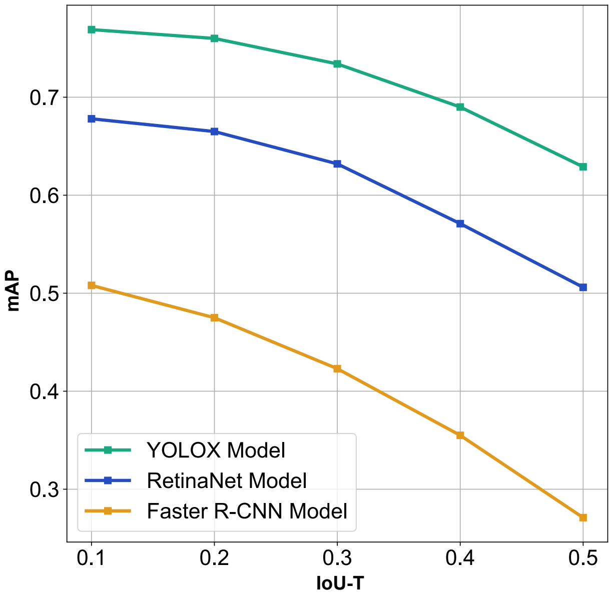

Supplementary Figure 5 Performance comparison between the Faster R-CNN model, the RetinaNet model and the YOLOX model on the held-out test dataset with different IoU-Ts.

mAP: mean average precision, IoU-T: intersection over union threshold.

(Source data are provided as a Source Data file.)

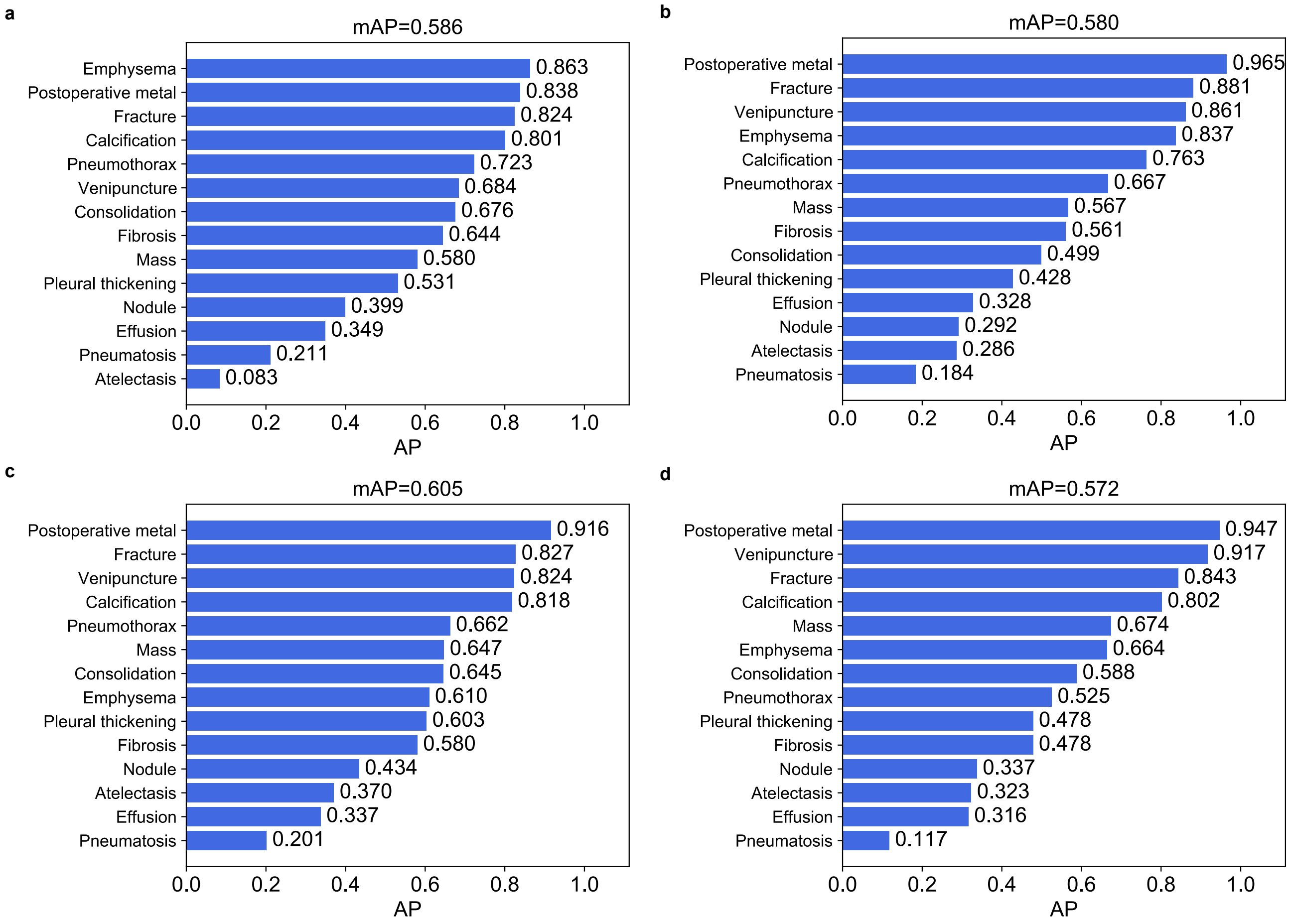

Supplementary Figure 6 Performance of the YOLOX model on the multicentre datasets with an IoU-T of 0.5

a External dataset A, **b** External dataset B, **c** External dataset C, and **d** External dataset D. mAP: mean average precision. (Source data are provided as a Source Data file.)

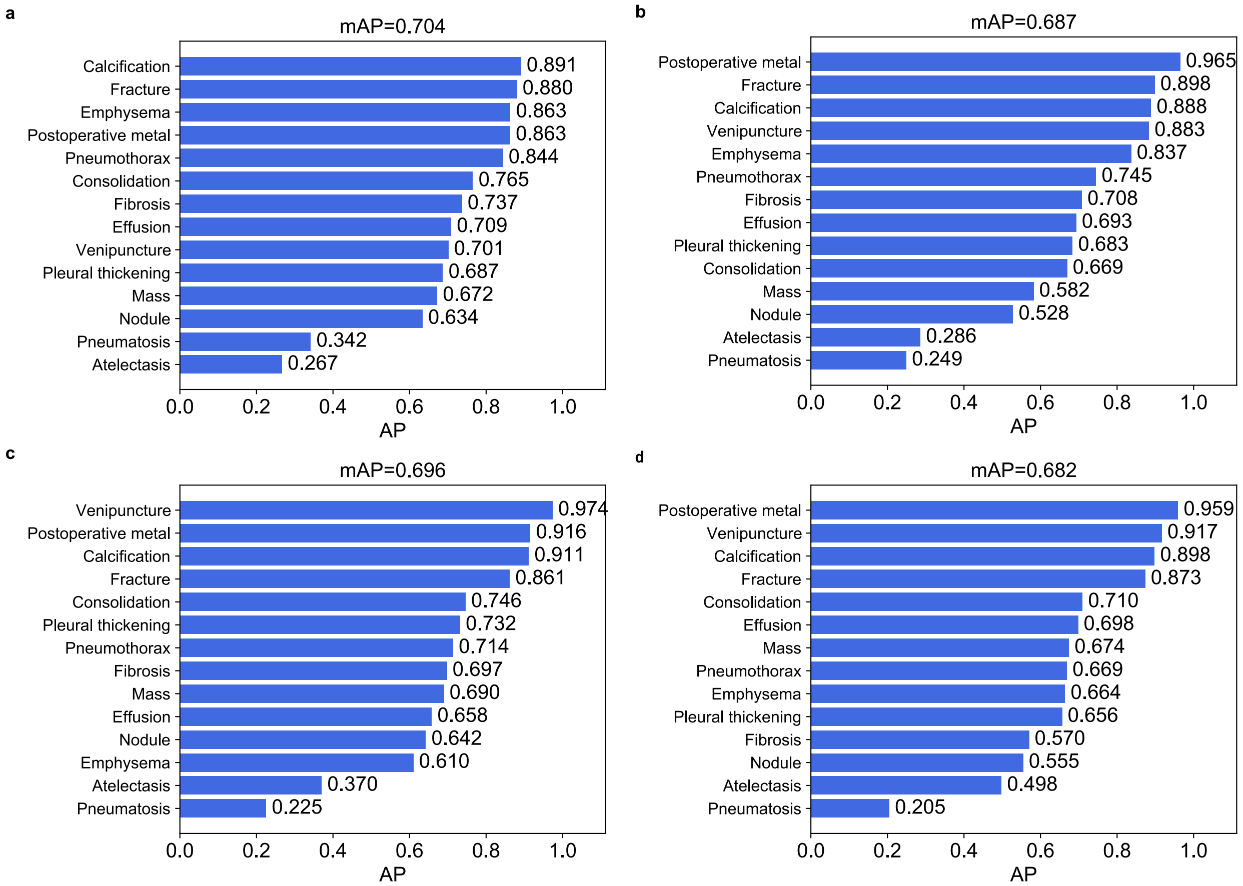

Supplementary Figure 7 Performance of the YOLOX model on the multicentre datasets with an IoU-T of 0.3
a External dataset A, **b** External dataset B, **c** External dataset C, and **d** External dataset D. mAP: mean average precision. (Source data are provided as a Source Data file.)

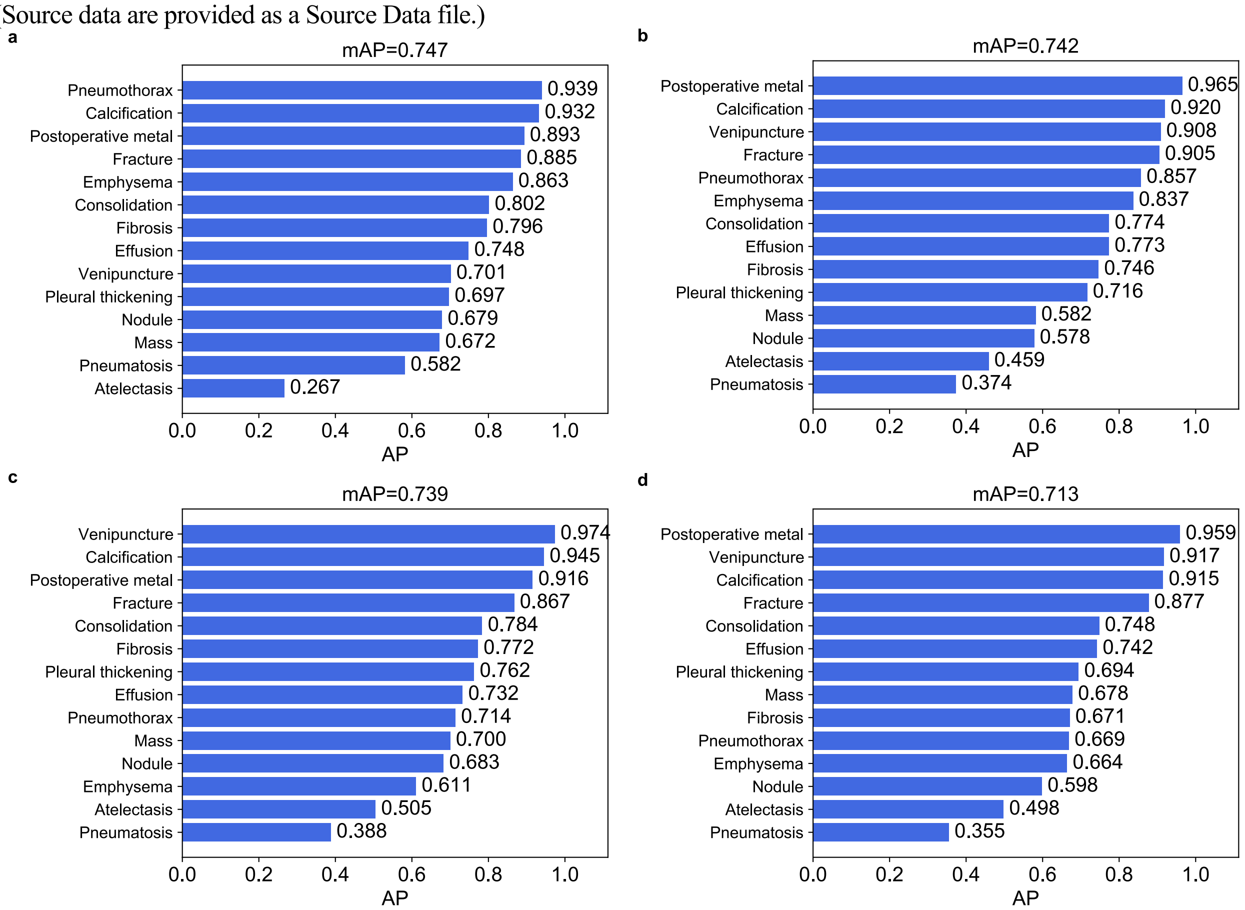

Supplementary Figure 8 Performance of the YOLOX model on the multicentre datasets with an IoU-T of 0.1
a External dataset A, **b** External dataset B, **c** External dataset C, and **d** External dataset D. mAP: mean average precision. (Source data are provided as a Source Data file.)

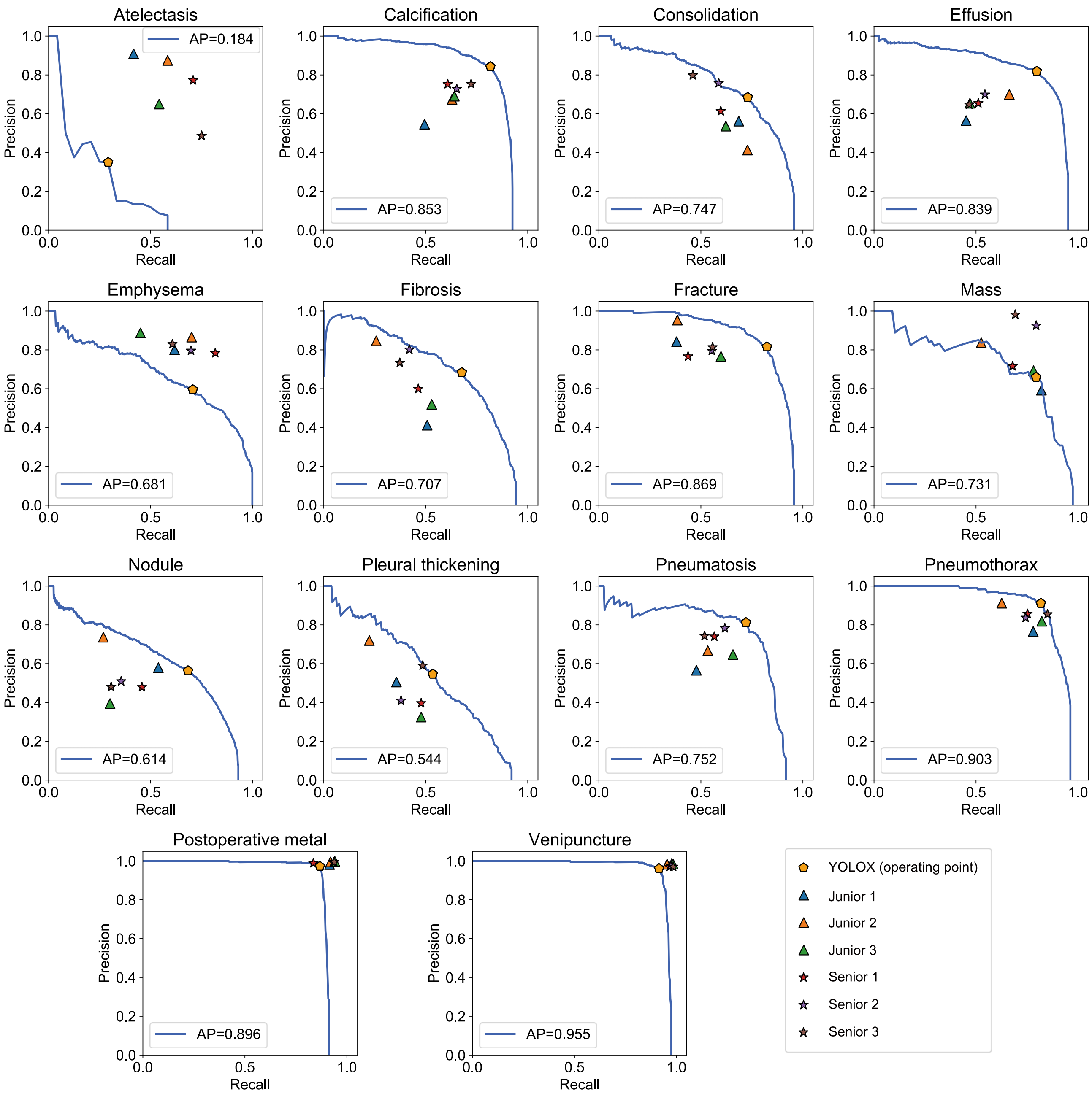

Supplementary Figure 9 Performance comparison between the YOLOX model and radiologists on the held-out test dataset with an IoU-T of 0.3.

The triangles represent the junior radiologists, the pentacles represent the senior radiologists, and the pentagon represents the model operation point. AP: average precision. IoU-T: intersection over union threshold.

(Source data are provided as a Source Data file.)

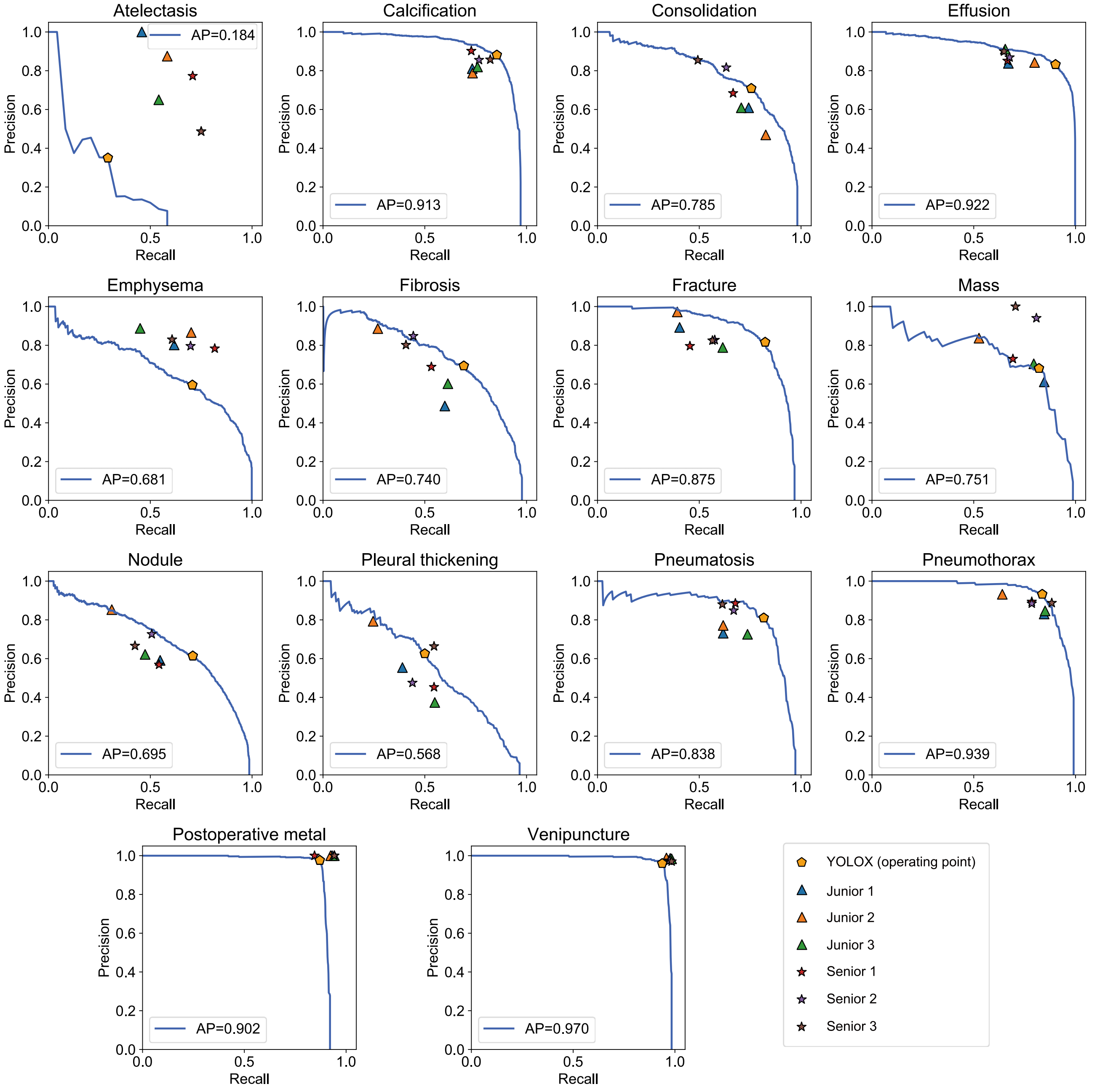

Supplementary Figure 10 Performance comparison between the YOLOX model and radiologists on the held-out test dataset with an IoU-T of 0.1.

The triangles represent the junior radiologists, the pentacles represent the senior radiologists, and the pentagon represents the model operation point. AP: average precision. IoU-T: intersection over union threshold.

(Source data are provided as a Source Data file.)

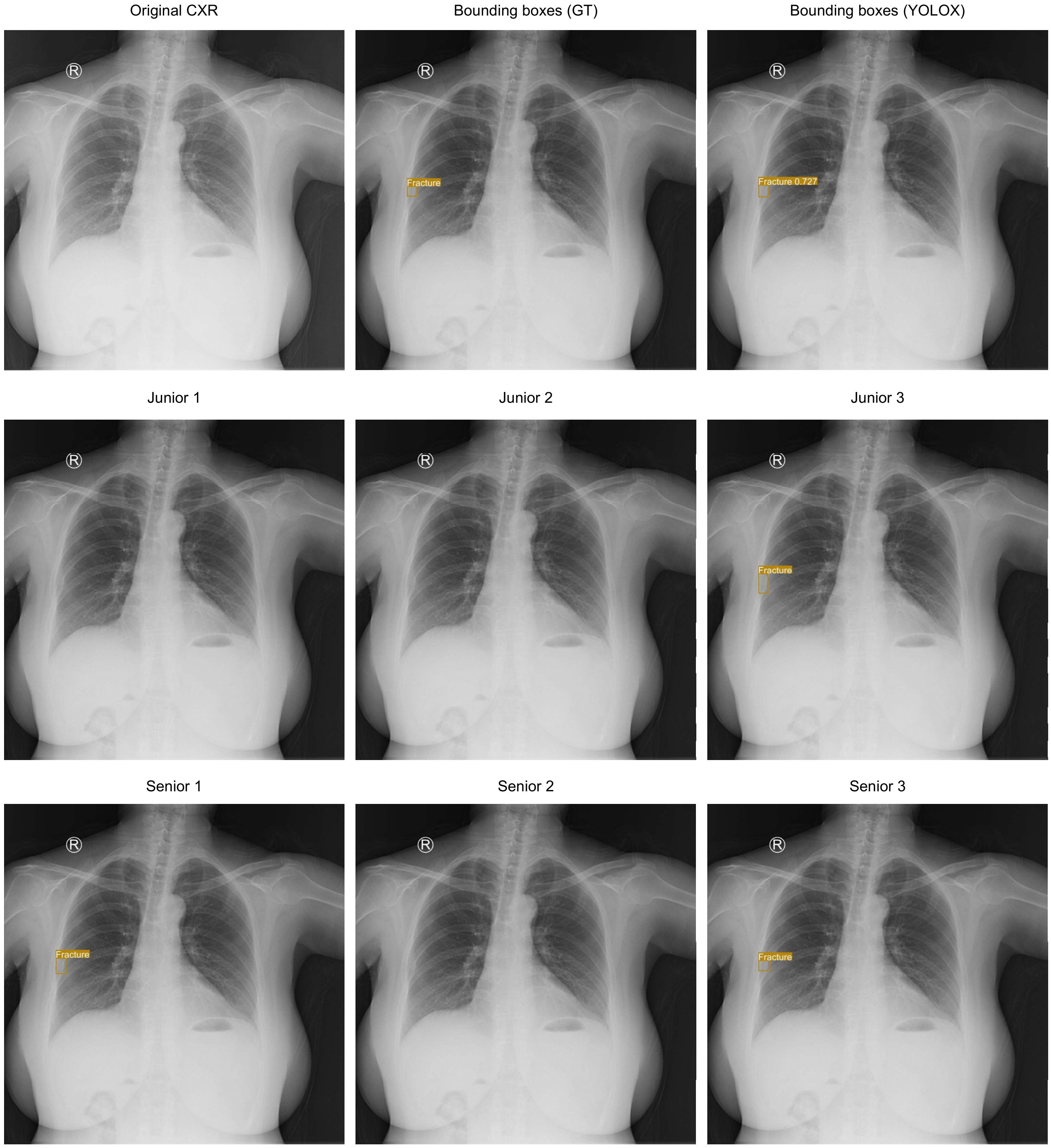

Supplementary Figure 11 A case of the YOLOX model and radiologists for the identification and localization of fracture in CXR.

The Bounding box of fracture obtained by the YOLOX model has a good consistency with GT Bounding box. But two junior radiologists and one senior radiologist missed the fracture when interpreting the CXR. GT: ground-truth.

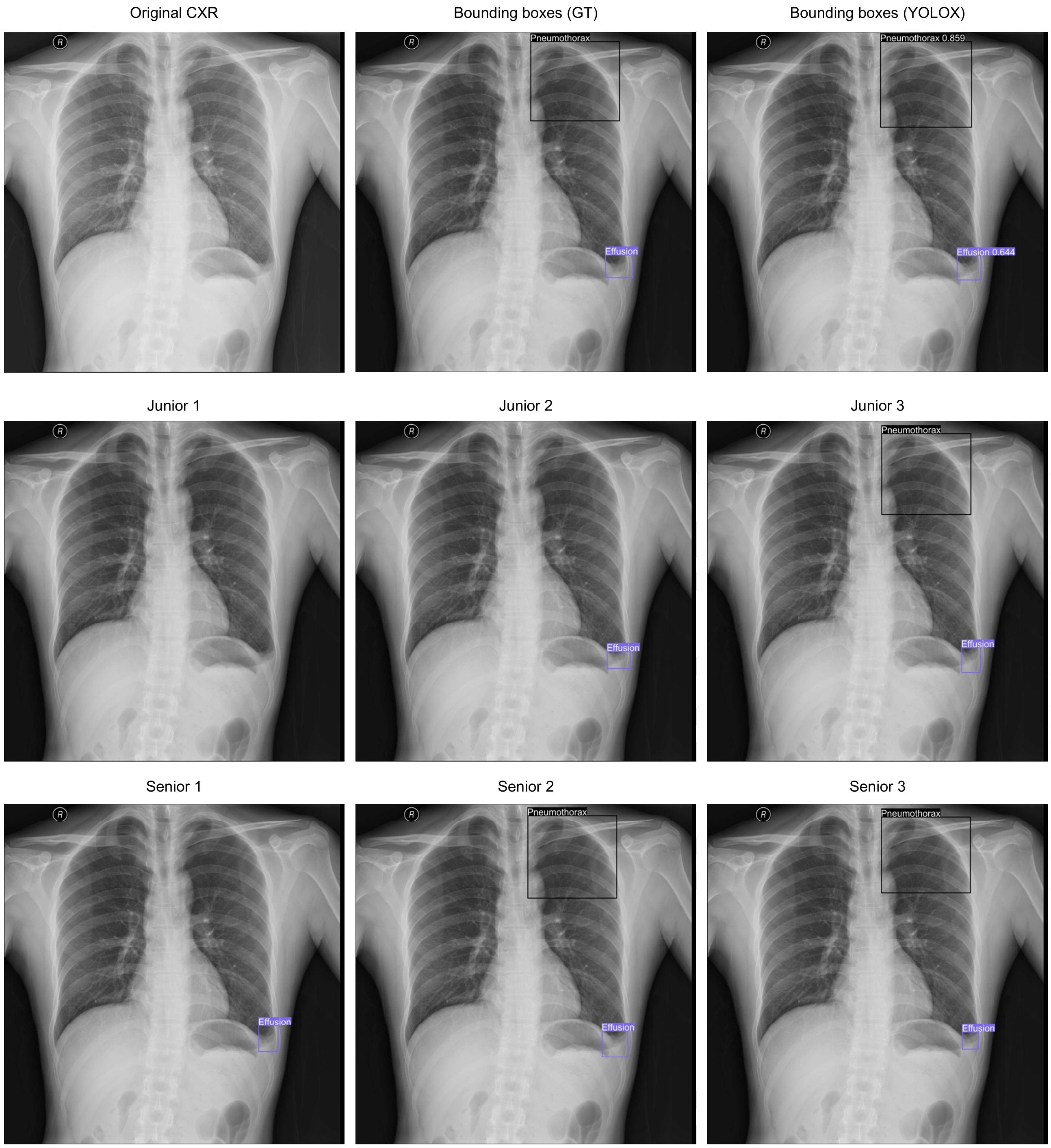

Supplementary Figure 12 A case of the YOLOX model and radiologists for the identification and localization of pneumothorax and effusion in CXR.

The Bounding boxes of pneumothorax and effusion obtained by the YOLOX model has a good consistency with GT Bounding boxes. But one junior radiologist missed the pneumothorax and effusion, one junior and one senior radiologist missed the pneumothorax when interpreting the CXR. GT: ground-truth.

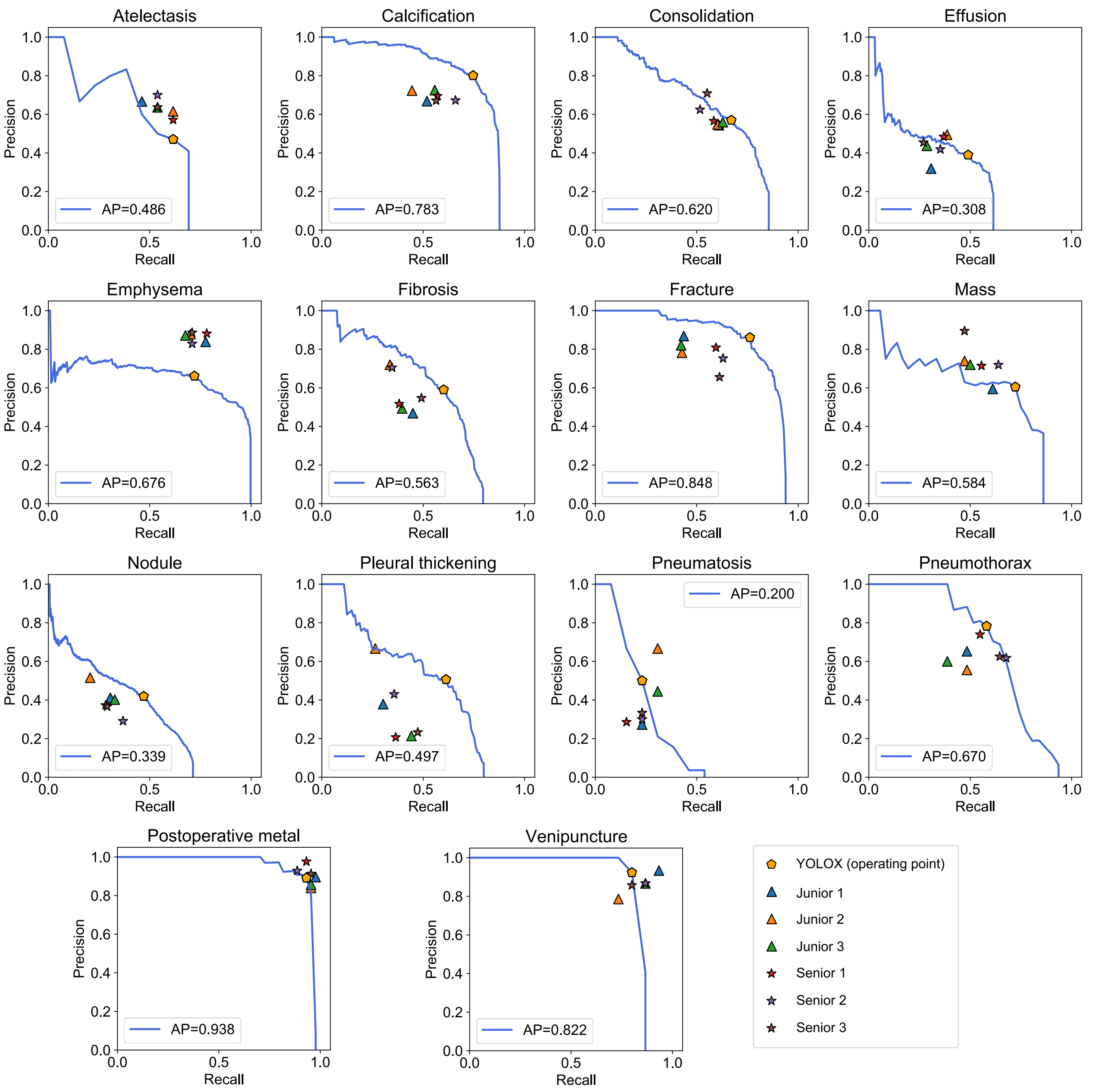

Supplementary Figure 13 Performance comparison between the YOLOX model and radiologists on the recombination test dataset with an IoU-T of 0.5.

The triangles represent the junior radiologists, the pentacles represent the senior radiologists, and the pentagon represents the model operation point. AP: average precision. IoU-T: intersection over union threshold.

(Source data are provided as a Source Data file.)

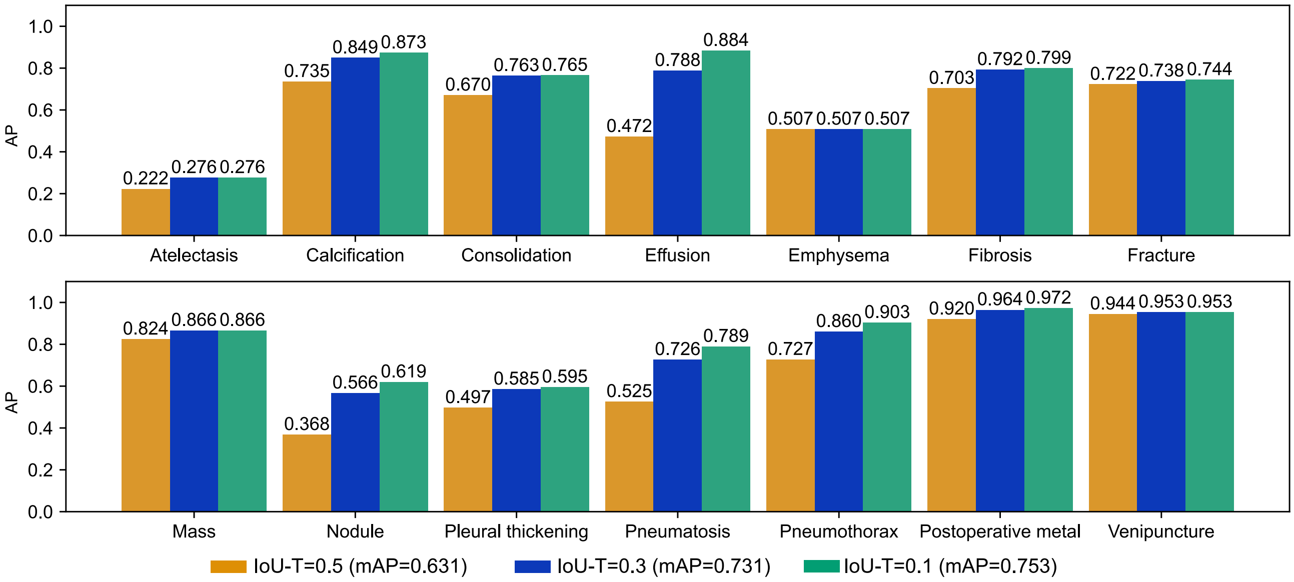

Supplementary Figure 14 Performance of the YOLOX model on the prospective dataset with different IoU-Ts. mAP: mean average precision, IoU-T: intersection over union threshold.

(Source data are provided as a Source Data file.)

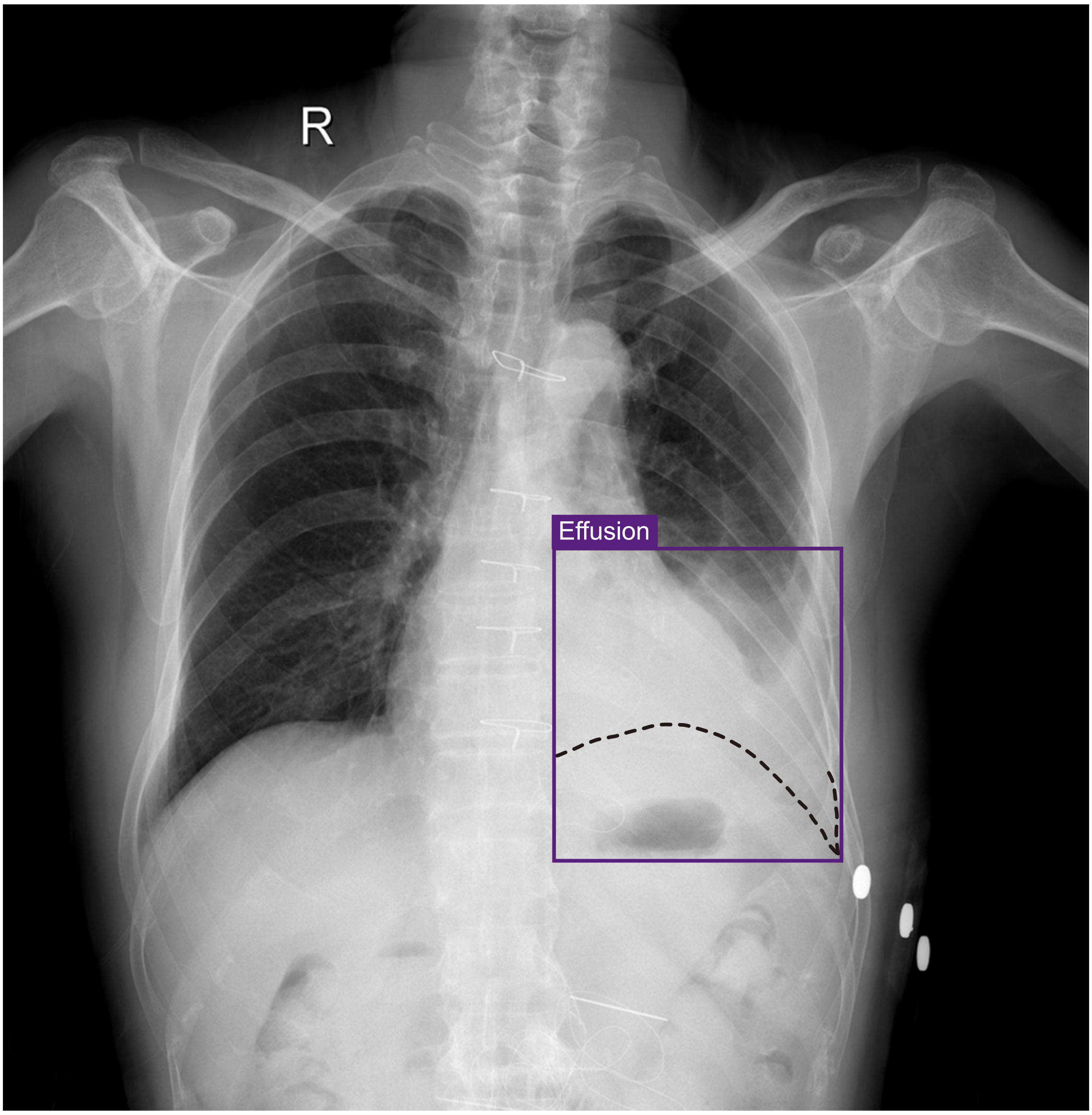

Supplementary Figure 15 An example to illustrate annotation rule 2.

In this CXR, a moderate left pleural effusion sheltered the left hemidiaphragm and costophrenic angle. Thus, the lower edge of the bounding box of the effusion cannot easily be determined. Radiologists should use their knowledge and experience to determine the position of the left hemidiaphragm and costophrenic angle by referring to their position on the right side (shown as the dotted line in the figure), and further annotate the lower boundary of the left pleural effusion.

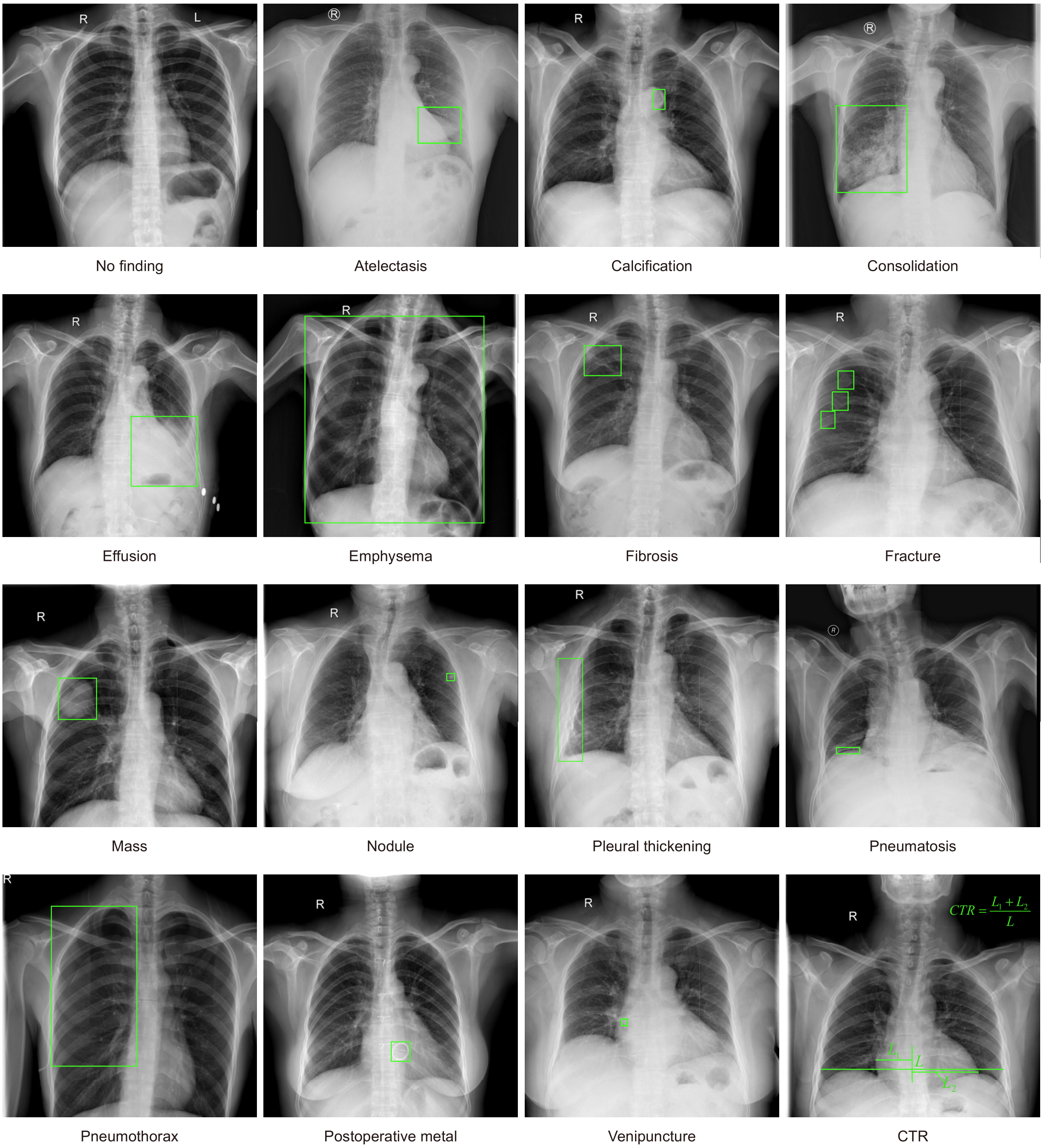

Supplementary Figure 16 Annotation examples of each abnormality and the calculation of the CTR for CXRs. CTR: cardiothoracic ratio.

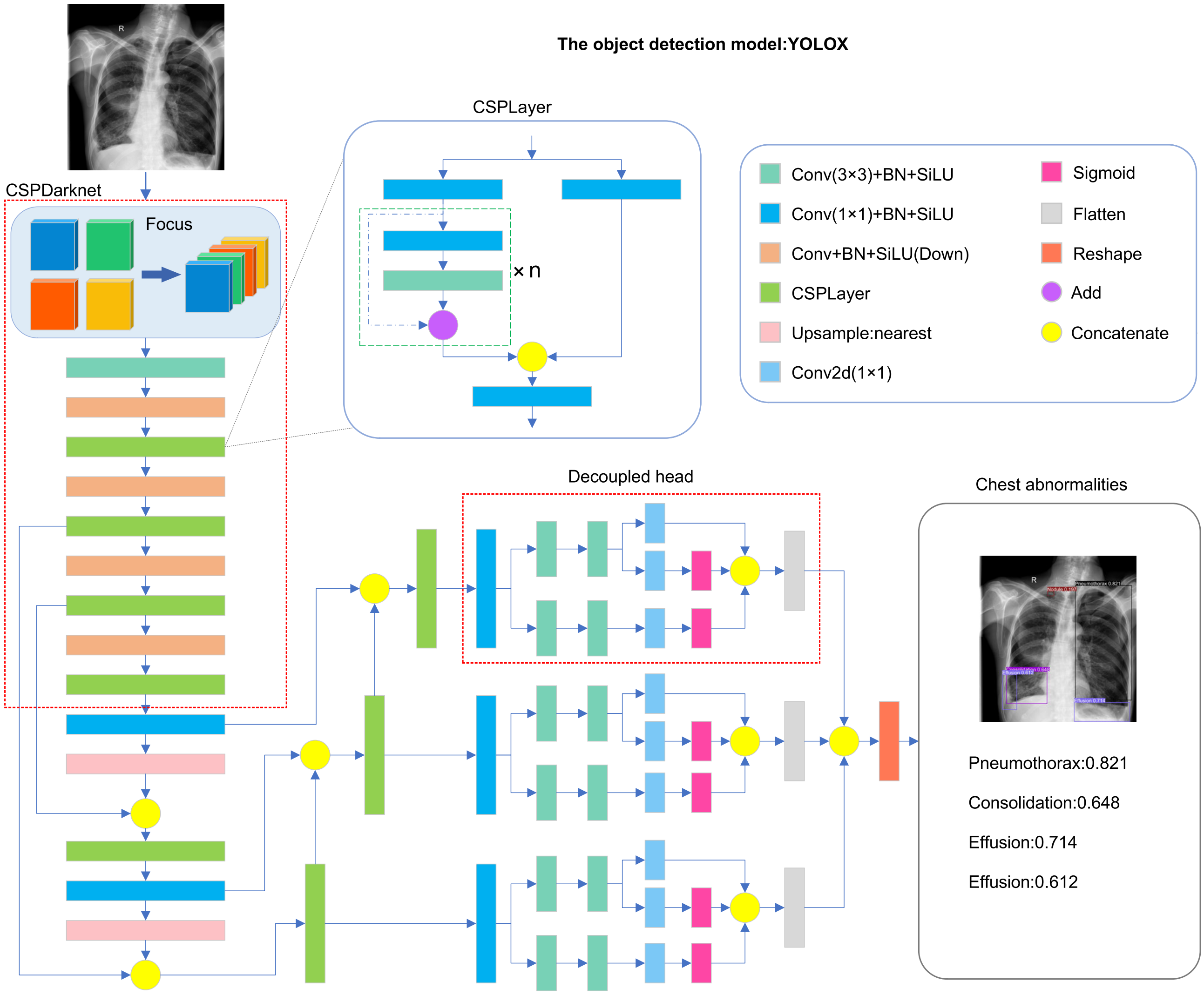

Supplementary Figure 17 The structure of the YOLOX model and its input and output.
Conv: convolution; BN: batch normalization; SiLU: the sigmoid-weighted activation function.

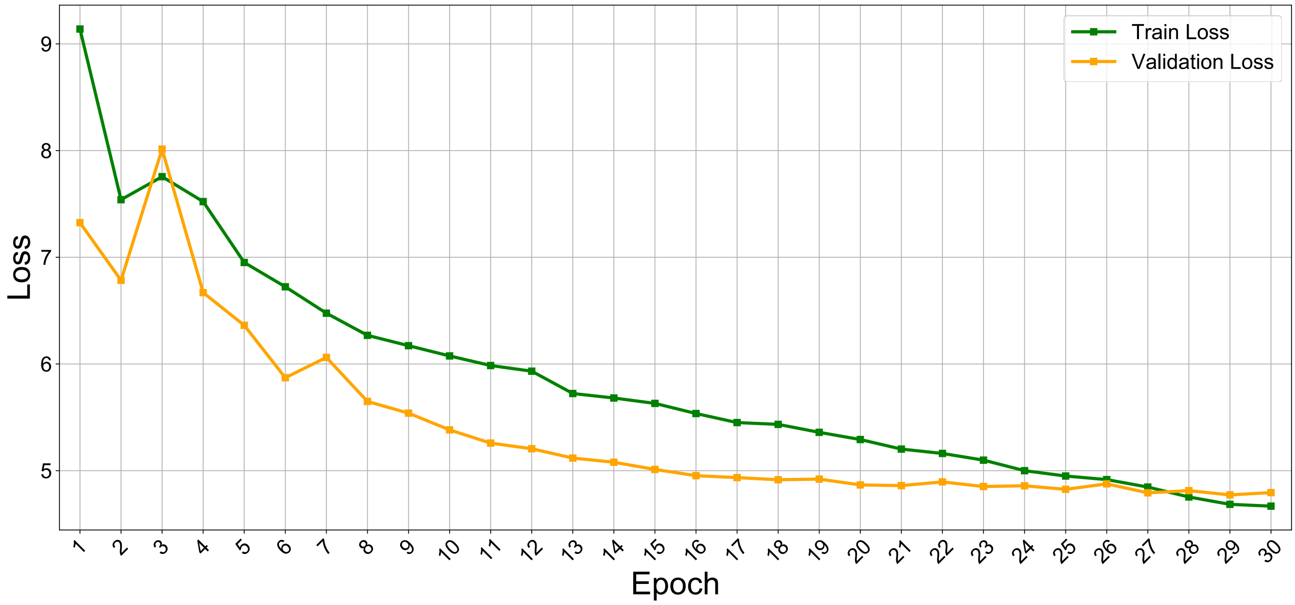

Supplementary Figure 18 Loss curves of the YOLOX model with different numbers of epochs.
(Source data are provided as a Source Data file.)

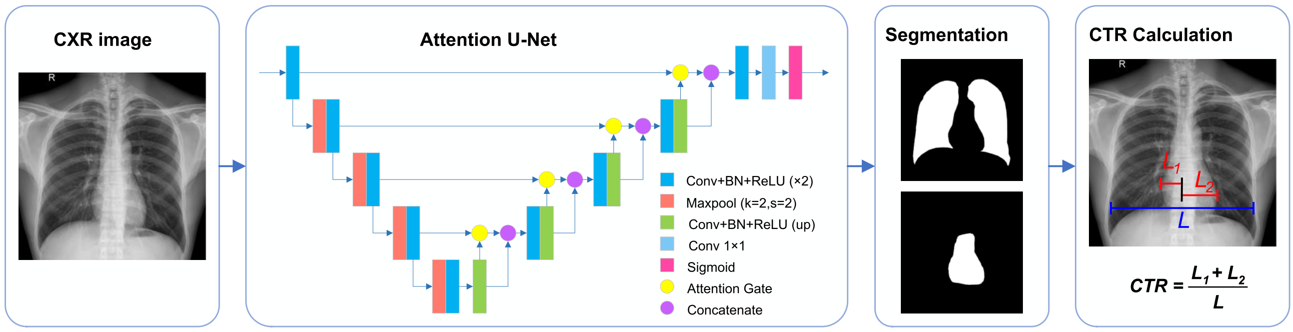

Supplementary Figure 19 Flowchart of the proposed CTR calculation algorithm.
Conv: convolution, BN: batch normalization, ReLU: the rectified linear unit activation function.

Supplementary Tables

Supplementary Table 1 Manufacturer and device information concerning the CXRs in each dataset.

Manufacturer information	Device information	CXR-AL14 dataset		Held-out test dataset	External dataset A	External dataset B	External dataset C	External dataset D	Prospective dataset
		Training dataset	Tuning dataset						
**Shimadzu**	**RADspeed Pro 50**	0	0	0	9	2628	2	0	0
**Carestream Health**	**DRX Evolution**	8685	947	415	9	0	718	0	0
**United-imaging Health**	**uDR 770i**	0	0	0	0	0	0	0	606
**KODAK**	**DirectView DR7500**	12286	1423	544	0	0	0	0	359
	**DirectView DR3500**	0	0	0	2371	0	0	0	0
	**DirectView DR3000**	0	0	0	0	0	1929	0	0
**SIEMENS**	**AXIOM Aristos VX Plus**	128454	14193	5041	0	0	0	0	575
	**Multix Fusion Max**	0	0	0	589	0	0	0	0
**DDIT**	**WV3000T**	0	0	0	0	5	0	0	0
**Mindray**	**DigiEye 680**	0	0	0	0	0	2	0	0
**General Medical Merate S.p.A.**	**CALPYSO**	0	0	0	0	0	0	2683	0
**Total**		149425	16563	6000	2978	2633	2651	2683	1540

(Source data are provided as a Source Data file.)

Supplementary Table 2 The IoU (mean ± SD) of all the bounding boxes for each abnormality on interreader or intrareader variability

		Atelectasis	Calcification	Consolidation	Effusion	Emphysema	Fibrosis	Fracture	Mass	Nodule	Pleural thickening	Pneumatosis	Pneumothorax	Postoperative metal	Venipuncture
**Inter-reader (mean ±SD)**	**E 1 vs E 2**	0.896±0.048	0.767±0.098	0.864±0.072	0.813±0.103	0.967±0.021	0.809±0.102	0.793±0.095	0.838±0.071	0.800±0.113	0.820±0.067	0.850±0.080	0.905±0.059	0.864±0.089	0.761±0.105
	**E 1 vs E 3**	0.826±0.030	0.730±0.103	0.801±0.094	0.771±0.106	0.944±0.022	0.755±0.094	0.736±0.088	0.859±0.085	0.733±0.107	0.799±0.081	0.820±0.101	0.880±0.057	0.835±0.078	0.748±0.096
	**E 1 vs E 4**	0.868±0.027	0.753±0.097	0.841±0.084	0.784±0.109	0.958±0.019	0.766±0.108	0.749±0.102	0.859±0.083	0.772±0.105	0.794±0.081	0.822±0.090	0.889±0.065	0.853±0.086	0.769±0.109
	**E 1 vs E 5**	0.849±0.065	0.742±0.113	0.822±0.084	0.801±0.099	0.950±0.023	0.789±0.099	0.760±0.092	0.882±0.037	0.773±0.113	0.792±0.087	0.812±0.105	0.880±0.061	0.855±0.078	0.743±0.101
	**E 1 vs E 6**	0.894±0.048	0.729±0.095	0.810±0.090	0.754±0.118	0.956±0.022	0.764±0.097	0.736±0.094	0.863±0.058	0.717±0.123	0.754±0.084	0.823±0.095	0.846±0.102	0.841±0.084	0.725±0.095
	**E 2 vs E 3**	0.868±0.017	0.699±0.104	0.802±0.101	0.772±0.109	0.944±0.025	0.744±0.116	0.791±0.071	0.872±0.069	0.748±0.108	0.807±0.074	0.831±0.094	0.900±0.071	0.812±0.080	0.779±0.083
	**E 2 vs E 4**	0.927±0.022	0.825±0.087	0.884±0.065	0.843±0.094	0.968±0.019	0.859±0.086	0.836±0.097	0.918±0.025	0.804±0.098	0.869±0.047	0.869±0.085	0.916±0.047	0.891±0.073	0.811±0.080
	**E 2 vs E 5**	0.911±0.044	0.803±0.105	0.875±0.089	0.842±0.104	0.956±0.023	0.867±0.085	0.842±0.091	0.829±0.084	0.805±0.094	0.852±0.103	0.871±0.080	0.915±0.061	0.895±0.073	0.787±0.082
	**E 2 vs E 6**	0.893±0.047	0.755±0.117	0.844±0.100	0.807±0.104	0.963±0.023	0.812±0.120	0.790±0.112	0.823±0.090	0.736±0.122	0.854±0.083	0.834±0.094	0.872±0.109	0.849±0.097	0.768±0.111
	**E 3 vs E 4**	0.867±0.054	0.701±0.113	0.784±0.098	0.729±0.118	0.947±0.023	0.705±0.113	0.750±0.093	0.883±0.057	0.762±0.098	0.775±0.074	0.788±0.124	0.884±0.068	0.814±0.075	0.748±0.073
	**E 3 vs E 5**	0.834±0.023	0.698±0.106	0.784±0.101	0.758±0.115	0.967±0.017	0.747±0.109	0.772±0.075	0.831±0.085	0.750±0.109	0.780±0.090	0.808±0.095	0.881±0.075	0.828±0.087	0.747±0.094
	**E 3 vs E 6**	0.825±0.034	0.714±0.107	0.785±0.101	0.723±0.129	0.936±0.026	0.733±0.107	0.737±0.088	0.857±0.084	0.755±0.110	0.766±0.083	0.804±0.109	0.853±0.114	0.855±0.085	0.739±0.076
	**E 4 vs E 5**	0.873±0.053	0.774±0.097	0.846±0.079	0.801±0.103	0.952±0.020	0.809±0.093	0.782±0.087	0.841±0.082	0.792±0.093	0.819±0.085	0.831±0.117	0.899±0.060	0.863±0.086	0.771±0.088
	**E 4 vs E 6**	0.860±0.034	0.749±0.119	0.848±0.091	0.805±0.106	0.964±0.021	0.809±0.116	0.751±0.113	0.843±0.067	0.739±0.120	0.820±0.084	0.831±0.101	0.872±0.112	0.852±0.089	0.776±0.124
	**E 5 vs E 6**	0.843±0.017	0.723±0.117	0.827±0.094	0.777±0.099	0.943±0.025	0.802±0.101	0.758±0.119	0.882±0.047	0.720±0.118	0.810±0.093	0.815±0.108	0.871±0.106	0.845±0.101	0.727±0.102
**Intra-reader (mean ±SD)**	**E 1 vs E 1***	0.931±0.041	0.804±0.109	0.905±0.087	0.864±0.098	0.983±0.010	0.833±0.111	0.791±0.115	0.929±0.021	0.773±0.087	0.857±0.080	0.858±0.116	0.948±0.035	0.863±0.110	0.806±0.074
	**E 2 vs E 2***	0.939±0.028	0.851±0.101	0.921±0.064	0.886±0.079	0.977±0.011	0.888±0.082	0.877±0.066	0.959±0.009	0.803±0.087	0.939±0.036	0.864±0.114	0.927±0.049	0.909±0.046	0.837±0.080
	**E 3 vs E 3***	0.947±0.019	0.839±0.102	0.924±0.072	0.897±0.076	0.985±0.007	0.891±0.088	0.880±0.086	0.934±0.069	0.817±0.089	0.914±0.049	0.910±0.094	0.957±0.040	0.928±0.057	0.773±0.096
	**E 4 vs E 4***	0.912±0.084	0.819±0.113	0.913±0.085	0.866±0.097	0.977±0.015	0.897±0.063	0.854±0.108	0.944±0.021	0.803±0.091	0.881±0.103	0.850±0.121	0.961±0.024	0.907±0.069	0.792±0.074
	**E 5 vs E 5***	0.973±0.016	0.897±0.078	0.941±0.076	0.925±0.093	0.992±0.007	0.940±0.045	0.878±0.099	0.976±0.008	0.824±0.089	0.924±0.093	0.858±0.139	0.979±0.013	0.944±0.033	0.855±0.059
	**E 6 vs E 6***	0.956±0.025	0.828±0.108	0.896±0.075	0.848±0.101	0.971±0.016	0.879±0.082	0.869±0.063	0.934±0.037	0.810±0.095	0.902±0.064	0.867±0.104	0.940±0.039	0.923±0.056	0.803±0.095

*. The twice annotations of each expert. IoU: intersection over union.

(Source data are provided as a Source Data file.)

Supplementary Table 3 Number of bounding boxes with IoU=0 for each abnormality on interreader or intrareader variability

		Atelectasis	Calcification	Consolidation	Effusion	Emphysema	Fibrosis	Fracture	Mass	Nodule	Pleural thickening	Pneumatosis	Pneumothorax	Postoperative metal	Venipuncture
Inter-reader (N=600)	**E 1 vs E 2**	0	1	2	0	0	2	0	0	3	0	0	0	0	0
	**E 1 vs E 3**	0	7	0	0	0	7	0	0	13	0	0	0	0	0
	**E 1 vs E 4**	0	2	2	0	0	2	0	0	10	0	0	0	0	0
	**E 1 vs E 5**	0	2	6	0	0	2	0	0	1	0	0	0	0	0
	**E 1 vs E 6**	0	4	3	0	0	2	0	0	11	0	0	0	0	0
	**E 2 vs E 3**	0	8	2	1	0	5	0	0	9	0	0	0	0	0
	**E 2 vs E 4**	0	0	0	0	0	0	0	0	7	0	0	0	0	0
	**E 2 vs E 5**	0	0	4	0	0	0	0	0	3	0	0	0	0	0
	**E 2 vs E 6**	0	2	1	0	0	0	0	0	9	0	0	0	0	0
	**E 3 vs E 4**	0	9	2	1	0	5	0	0	14	0	0	0	0	0
	**E 3 vs E 5**	0	9	6	1	0	5	0	0	12	0	0	0	0	0
	**E 3 vs E 6**	0	7	3	1	0	5	0	0	10	0	0	0	0	0
	**E 4 vs E 5**	0	0	4	0	0	0	0	0	10	0	0	0	0	0
	**E 4 vs E 6**	0	2	1	0	0	0	0	0	10	0	0	0	0	0
	**E 5 vs E 6**	0	2	5	0	0	0	0	0	10	0	0	0	0	0
Intra-reader (N=600)	**E 1 vs E 1***	0	1	1	1	0	0	1	0	2	0	0	0	0	0
	**E 2 vs E 2***	0	1	0	0	0	2	0	0	2	0	0	0	0	0
	**E 3 vs E 3***	0	0	1	0	0	2	0	0	2	0	1	0	0	0
	**E 4 vs E 4***	0	1	2	0	0	1	0	0	1	0	0	0	0	0
	**E 5 vs E 5***	0	1	1	0	0	4	1	0	6	1	0	0	0	0
	**E 6 vs E 6***	0	0	1	0	0	1	1	0	1	0	0	0	0	0

*. The twice annotations of each expert. IoU: intersection over union.

(Source data are provided as a Source Data file.)

Supplementary Table 4 General information and distribution of all abnormalities in the CXRs contained in each test dataset.

	Held-out test dataset	External dataset A	External dataset B	External dataset C	External dataset D	Recombination dataset	Prospective dataset
**Collected CXR**	6000	3314	3021	3294	2997	2800	2701
**Included CXR**	6000	2978	2633	2651	2683	2800	1540
**Abnormal CXR**	3692(61.533%)	1334(44.795%)	1304(49.525%)	1934(72.954%)	1548(57.697%)	1615(57.679%)	946(61.429%)
**No-Finding CXR**	2308(38.467%)	1644(55.205%)	1329(50.475%)	717(27.046%)	1135(42.303%)	1185(42.321%)	594(38.571%)
**Patients**	5946	2978	2617	2631	2662	2797	1517
**Age (mean ±sd)**	53.56±15.00	48.43±17.96	44.27±17.60	58.42±15.33	53.27±22.71	51.57±18.44	54.73±15.03
**Gender (males, %)**	3427(57.117%)	1343(45.097%)	1320(50.133%)	1551(58.506%)	1413(52.665%)	1427(50.964%)	759(49.286%)
**Collection time**	2011.08-2021.12	2021.01-2021.12	2021.08-2022.03	2014.01-2022.03	2015.07-2020.05	2015.07-2022.03	2023.10-2023.10
**Atelectasis**	24(0.270%)	3(0.112%)	5(0.187%)	8(0.154%)	12(0.352%)	13(0.352%)	6(0.282%)
**Calcification**	1267(14.274%)	506(18.973%)	465(17.416%)	785(15.067%)	541(15.879%)	598(16.184%)	326(15.334%)
**Consolidation**	773(8.709%)	272(10.199%)	273(10.225%)	708(13.589%)	303(8.893%)	428(11.583%)	170(7.996%)
**Effusion**	1506(16.967%)	178(6.674%)	159(5.955%)	305(5.854%)	217(6.369%)	241(6.522%)	340(15.992%)
**Emphysema**	682(7.684%)	309(11.586%)	211(7.903%)	433(8.311%)	317(9.304%)	361(9.770%)	208(9.784%)
**Fibrosis**	665(7.492%)	204(7.649%)	176(6.592%)	513(9.846%)	158(4.638%)	283(7.659%)	149(7.008%)
**Fracture**	533(6.005%)	146(5.474%)	151(5.655%)	244(4.683%)	316(9.275%)	227(6.143%)	104(4.892%)
**Mass**	78(0.879%)	18(0.675%)	22(0.824%)	59(1.132%)	26(0.763%)	36(0.974%)	23(1.082%)
**Nodule**	1528(17.215%)	814(30.521%)	1027(38.464%)	1890(36.276%)	1331(39.067%)	1276(34.533%)	253(11.900%)
**Pleural thickening**	264(2.974%)	70(2.625%)	97(3.633%)	165(3.167%)	90(2.642%)	129(3.491%)	95(4.468%)
**Pneumatosis**	251(2.828%)	20(0.750%)	12(0.449%)	18(0.345%)	24(0.704%)	13(0.352%)	120(5.644%)
**Pneumothorax**	214(2.411%)	17(0.637%)	24(0.899%)	50(0.960%)	20(0.587%)	31(0.839%)	78(3.669%)
**Postoperative Metal**	658(7.413%)	95(3.562%)	29(1.086%)	15(0.288%)	49(1.438%)	44(1.191%)	175(8.231%)
**Venipuncture**	433(4.878%)	15(0.562%)	19(0.712%)	17(0.326%)	3(0.088%)	15(0.406%)	79(3.716%)
**Total**	8876	2667	2670	5210	3407	3695	2126

(Source data are provided as a Source Data file.)

Supplementary Table 5 The AP values of each abnormality achieved by the Faster R-CNN model, the RetinaNet model and the YOLOX model on the held-out test dataset with different IoU-Ts.

	IoU-T=0.5			IoU-T=0.3			IoU-T=0.1		
	Faster R-CNN	RetinaNet	YOLOX	Faster R-CNN	RetinaNet	YOLOX	Faster R-CNN	RetinaNet	YOLOX
**Atelectasis**	0.275	0.028	0.152	0.318	0.145	0.184	0.318	0.153	0.184
**Calcification**	0.061	0.637	0.725	0.356	0.792	0.853	0.609	0.859	0.913
**Consolidation**	0.470	0.455	0.599	0.659	0.615	0.747	0.734	0.683	0.785
**Effusion**	0.321	0.499	0.627	0.699	0.783	0.839	0.864	0.875	0.922
**Emphysema**	0.744	0.731	0.681	0.744	0.731	0.681	0.744	0.731	0.681
**Fibrosis**	0.143	0.431	0.601	0.337	0.567	0.707	0.422	0.605	0.740
**Fracture**	0.014	0.637	0.819	0.075	0.713	0.869	0.108	0.725	0.875
**Mass**	0.531	0.546	0.718	0.613	0.592	0.731	0.666	0.623	0.751
**Nodule**	0.006	0.474	0.348	0.035	0.495	0.614	0.095	0.503	0.695
**Pleural thickening**	0.178	0.205	0.407	0.334	0.337	0.544	0.387	0.376	0.568
**Pneumatosis**	0.152	0.214	0.551	0.379	0.536	0.752	0.518	0.706	0.838
**Pneumothorax**	0.479	0.579	0.792	0.595	0.745	0.903	0.693	0.812	0.939
**Postoperative Metal**	0.418	0.875	0.867	0.739	0.943	0.896	0.850	0.958	0.902
**Venipuncture**	0.008	0.777	0.916	0.037	0.856	0.955	0.108	0.887	0.970
**mAP**	0.271	0.506	0.629	0.423	0.632	0.734	0.508	0.678	0.769

mAP: mean average precision, IoU-T: intersection over union threshold.

(Source data are provided as a Source Data file.)

Supplementary Table 6 Precision, recall and F1-score values achieved by the YOLOX model on the multicentre and prospective datasets with an IoU-T of 0.5

	External dataset A			External dataset B			External dataset C			External dataset D			Prospective dataset		
	**Precision**	**Recall**	**F1**	**Precision**	**Recall**	**F1**	**Precision**	**Recall**	**F1**	**Precision**	**Recall**	**F1**	**Precision**	**Recall**	**F1**
Atelectasis	0.250 (0.046-0.699)	0.333 (0.061-0.792)	0.286	0.667 (0.208-0.939)	0.400 (0.118-0.769)	0.500	0.500 (0.215-0.785)	0.500 (0.215-0.785)	0.500	0.500 (0.237-0.763)	0.417 (0.193-0.680)	0.455	0.667 (0.208-0.939)	0.333 (0.097-0.700)	0.444
Calcification	0.816 (0.779-0.848)	0.789 (0.751-0.822)	0.802	0.798 (0.758-0.833)	0.757 (0.716-0.794)	0.777	0.756 (0.727-0.784)	0.823 (0.795-0.848)	0.788	0.775 (0.737-0.808)	0.756 (0.718-0.790)	0.765	0.763 (0.714-0.806)	0.761 (0.712-0.804)	0.762
Consolidation	0.703 (0.647-0.754)	0.706 (0.649-0.757)	0.705	0.508 (0.454-0.562)	0.604 (0.545-0.661)	0.552	0.585 (0.552-0.618)	0.705 (0.670-0.737)	0.639	0.583 (0.530-0.634)	0.650 (0.595-0.702)	0.615	0.746 (0.669-0.811)	0.624 (0.549-0.693)	0.679
Effusion	0.430 (0.371-0.491)	0.618 (0.545-0.686)	0.507	0.433 (0.367-0.501)	0.566 (0.488-0.641)	0.490	0.423 (0.373-0.474)	0.502 (0.446-0.557)	0.459	0.408 (0.348-0.471)	0.452 (0.387-0.518)	0.429	0.601 (0.547-0.653)	0.576 (0.523-0.628)	0.589
Emphysema	0.808 (0.760-0.848)	0.790 (0.741-0.831)	0.799	0.715 (0.654-0.769)	0.796 (0.737-0.845)	0.753	0.523 (0.485-0.560)	0.820 (0.781-0.853)	0.638	0.492 (0.452-0.533)	0.915 (0.879-0.941)	0.640	0.573 (0.502-0.641)	0.529 (0.461-0.596)	0.550
Fibrosis	0.679 (0.610-0.741)	0.642 (0.574-0.705)	0.660	0.649 (0.569-0.721)	0.545 (0.472-0.617)	0.593	0.702 (0.654-0.746)	0.519 (0.475-0.561)	0.596	0.597 (0.511-0.678)	0.487 (0.411-0.565)	0.537	0.766 (0.685-0.831)	0.658 (0.578-0.729)	0.708
Fracture	0.759 (0.687-0.819)	0.822 (0.752-0.875)	0.789	0.875 (0.809-0.920)	0.788 (0.716-0.846)	0.829	0.739 (0.682-0.788)	0.799 (0.744-0.845)	0.768	0.818 (0.770-0.857)	0.766 (0.716-0.809)	0.791	0.835 (0.742-0.899)	0.683 (0.588-0.764)	0.751
Mass	0.750 (0.468-0.911)	0.500 (0.290-0.710)	0.600	0.542 (0.351-0.721)	0.591 (0.387-0.767)	0.565	0.600 (0.479-0.710)	0.661 (0.534-0.769)	0.629	0.537 (0.387-0.679)	0.846 (0.665-0.939)	0.657	0.810 (0.600-0.923)	0.739 (0.535-0.875)	0.773
Nodule	0.490 (0.456-0.524)	0.500 (0.466-0.534)	0.495	0.383 (0.354-0.413)	0.390 (0.361-0.421)	0.387	0.476 (0.455-0.497)	0.550 (0.527-0.572)	0.510	0.397 (0.373-0.421)	0.479 (0.453-0.506)	0.434	0.533 (0.463-0.602)	0.411 (0.352-0.473)	0.464
Pleural thickening	0.655 (0.523-0.766)	0.514 (0.400-0.628)	0.576	0.536 (0.437-0.632)	0.536 (0.437-0.632)	0.536	0.632 (0.553-0.704)	0.582 (0.506-0.654)	0.606	0.582 (0.472-0.685)	0.511 (0.410-0.612)	0.544	0.710 (0.587-0.808)	0.463 (0.366-0.563)	0.561
Pneumatosis	0.385 (0.177-0.645)	0.250 (0.112-0.469)	0.303	0.375 (0.137-0.694)	0.250 (0.089-0.532)	0.300	0.455 (0.213-0.720)	0.278 (0.125-0.509)	0.345	0.200 (0.095-0.373)	0.250 (0.120-0.449)	0.222	0.699 (0.599-0.783)	0.542 (0.453-0.628)	0.610
Pneumothorax	0.737 (0.512-0.882)	0.824 (0.590-0.938)	0.778	0.696 (0.491-0.844)	0.667 (0.467-0.820)	0.681	0.795 (0.645-0.892)	0.620 (0.482-0.741)	0.697	0.600 (0.387-0.781)	0.600 (0.387-0.781)	0.600	0.841 (0.737-0.909)	0.744 (0.637-0.827)	0.789
Postoperative Metal	0.862 (0.778-0.917)	0.853 (0.768-0.910)	0.857	0.933 (0.787-0.982)	0.966 (0.828-0.994)	0.949	0.933 (0.702-0.988)	0.933 (0.702-0.988)	0.933	1.000 (0.918-1.000)	0.878 (0.758-0.943)	0.935	0.914 (0.863-0.947)	0.909 (0.857-0.943)	0.911
Venipuncture	0.900 (0.596-0.982)	0.600 (0.357-0.802)	0.720	1.000 (0.806-1.000)	0.842 (0.624-0.945)	0.914	1.000 (0.785-1.000)	0.824 (0.590-0.938)	0.903	0.750 (0.301-0.954)	1.000 (0.439-1.000)	0.857	0.959 (0.886-0.986)	0.886 (0.797-0.939)	0.921
Mean	0.659	0.624	0.634	0.651	0.621	0.630	0.651	0.651	0.644	0.588	0.643	0.606	0.744	0.633	0.679

The 95% Wilson confidence interval was applied to both the precision and recall of different abnormality on each test dataset. IoU-T: intersection over union threshold.

(Source data are provided as a Source Data file.)

Supplementary Table 7 Precision, recall and F1-score values achieved by the YOLOX model on the multicentre and prospective datasets with an IoU-T of 0.3

	External dataset A			External dataset B			External dataset C			External dataset D			Prospective dataset		
	**Precision**	**Recall**	**F1**	**Precision**	**Recall**	**F1**	**Precision**	**Recall**	**F1**	**Precision**	**Recall**	**F1**	**Precision**	**Recall**	**F1**
**Atelectasis**	0.400 (0.118-0.769)	0.667 (0.208-0.939)	0.500	0.667 (0.208-0.939)	0.400 (0.118-0.769)	0.500	0.500 (0.215-0.785)	0.500 (0.215-0.785)	0.500	0.600 (0.313-0.832)	0.500 (0.254-0.746)	0.545	0.667 (0.208-0.939)	0.333 (0.097-0.700)	0.444
**Calcification**	0.873 (0.841-0.900)	0.844 (0.810-0.873)	0.858	0.864 (0.829-0.893)	0.819 (0.782-0.852)	0.841	0.814 (0.786-0.838)	0.885 (0.861-0.906)	0.848	0.830 (0.796-0.859)	0.821 (0.786-0.851)	0.825	0.814 (0.769-0.852)	0.834 (0.790-0.871)	0.824
**Consolidation**	0.755 (0.700-0.802)	0.757 (0.703-0.804)	0.756	0.611 (0.557-0.663)	0.714 (0.658-0.765)	0.659	0.640 (0.607-0.672)	0.771 (0.739-0.801)	0.700	0.636 (0.585-0.685)	0.739 (0.687-0.785)	0.684	0.714 (0.643-0.776)	0.735 (0.664-0.796)	0.725
**Effusion**	0.621 (0.559-0.678)	0.882 (0.826-0.922)	0.729	0.620 (0.553-0.683)	0.811 (0.743-0.865)	0.703	0.599 (0.548-0.649)	0.711 (0.658-0.759)	0.651	0.617 (0.557-0.674)	0.742 (0.680-0.796)	0.674	0.729 (0.682-0.772)	0.800 (0.754-0.839)	0.763
**Emphysema**	0.808 (0.760-0.848)	0.790 (0.741-0.831)	0.799	0.715 (0.654-0.769)	0.796 (0.737-0.845)	0.753	0.523 (0.485-0.560)	0.820 (0.781-0.853)	0.638	0.492 (0.452-0.533)	0.915 (0.879-0.941)	0.640	0.573 (0.502-0.641)	0.529 (0.461-0.596)	0.550
**Fibrosis**	0.733 (0.667-0.790)	0.701 (0.635-0.760)	0.717	0.736 (0.660-0.801)	0.619 (0.546-0.688)	0.673	0.759 (0.714-0.798)	0.583 (0.540-0.625)	0.659	0.678 (0.589-0.756)	0.506 (0.429-0.583)	0.580	0.812 (0.736-0.871)	0.698 (0.620-0.766)	0.751
**Fracture**	0.853 (0.784-0.903)	0.795 (0.722-0.852)	0.823	0.882 (0.817-0.926)	0.795 (0.723-0.851)	0.836	0.757 (0.702-0.804)	0.828 (0.776-0.870)	0.791	0.838 (0.792-0.876)	0.772 (0.723-0.815)	0.804	0.847 (0.756-0.908)	0.692 (0.598-0.773)	0.762
**Mass**	0.688 (0.444-0.858)	0.611 (0.386-0.797)	0.647	0.542 (0.351-0.721)	0.591 (0.387-0.767)	0.565	0.631 (0.509-0.738)	0.695 (0.569-0.797)	0.661	0.537 (0.387-0.679)	0.846 (0.665-0.939)	0.657	0.818 (0.615-0.927)	0.783 (0.581-0.903)	0.800
**Nodule**	0.631 (0.597-0.663)	0.646 (0.613-0.678)	0.638	0.528 (0.498-0.558)	0.547 (0.517-0.577)	0.537	0.586 (0.565-0.606)	0.688 (0.667-0.708)	0.633	0.513 (0.488-0.537)	0.619 (0.593-0.645)	0.561	0.617 (0.553-0.677)	0.573 (0.512-0.633)	0.594
**Pleural thickening**	0.620 (0.510-0.719)	0.700 (0.585-0.795)	0.658	0.701 (0.604-0.783)	0.701 (0.604-0.783)	0.701	0.647 (0.575-0.712)	0.721 (0.648-0.784)	0.682	0.675 (0.566-0.768)	0.600 (0.497-0.695)	0.635	0.758 (0.638-0.848)	0.495 (0.396-0.594)	0.599
**Pneumatosis**	0.438 (0.231-0.668)	0.350 (0.181-0.567)	0.389	0.333 (0.138-0.609)	0.333 (0.138-0.609)	0.333	0.455 (0.213-0.720)	0.278 (0.125-0.509)	0.345	0.304 (0.156-0.509)	0.292 (0.149-0.492)	0.298	0.840 (0.753-0.901)	0.658 (0.570-0.737)	0.738
**Pneumothorax**	0.929 (0.685-0.987)	0.765 (0.527-0.904)	0.839	0.739 (0.535-0.875)	0.708 (0.508-0.851)	0.723	0.821 (0.673-0.910)	0.640 (0.501-0.759)	0.719	0.700 (0.481-0.855)	0.700 (0.481-0.855)	0.700	0.913 (0.823-0.960)	0.808 (0.707-0.880)	0.857
**Postoperative Metal**	0.862 (0.778-0.917)	0.853 (0.768-0.910)	0.857	0.933 (0.787-0.982)	0.966 (0.828-0.994)	0.949	0.933 (0.702-0.988)	0.933 (0.702-0.988)	0.933	1.000 (0.918-1.000)	0.878 (0.758-0.943)	0.935	0.919 (0.871-0.950)	0.971 (0.935-0.988)	0.944
**Venipuncture**	0.900 (0.596-0.982)	0.600 (0.357-0.802)	0.720	1.000 (0.806-1.000)	0.842 (0.624-0.945)	0.914	0.850 (0.640-0.948)	1.000 (0.816-1.000)	0.919	0.750 (0.301-0.954)	1.000 (0.439-1.000)	0.857	0.973 (0.906-0.992)	0.899 (0.813-0.948)	0.934
**Mean**	0.722	0.711	0.709	0.705	0.689	0.692	0.68	0.718	0.691	0.655	0.709	0.671	0.785	0.701	0.735

The 95% Wilson confidence interval was applied to both the precision and recall of different abnormality on each test dataset. IoU-T: intersection over union threshold.

(Source data are provided as a Source Data file.)

Supplementary Table 8 Precision, recall and F1-score values achieved by the YOLOX model on the multicentre and prospective datasets with an IoU-T of 0.1

	External dataset A			External dataset B			External dataset C			External dataset D			Prospective dataset		
	**Precision**	**Recall**	**F1**	**Precision**	**Recall**	**F1**	**Precision**	**Recall**	**F1**	**Precision**	**Recall**	**F1**	**Precision**	**Recall**	**F1**
Atelectasis	0.400 (0.118-0.769)	0.667 (0.208-0.939)	0.500	0.400 (0.168-0.687)	0.800 (0.376-0.964)	0.533	0.625 (0.306-0.863)	0.625 (0.306-0.863)	0.625	0.600 (0.313-0.832)	0.500 (0.254-0.746)	0.545	0.667 (0.208-0.939)	0.333 (0.097-0.700)	0.444
Calcification	0.898 (0.868-0.922)	0.868 (0.835-0.894)	0.882	0.880 (0.846-0.907)	0.834 (0.798-0.865)	0.857	0.834 (0.807-0.857)	0.907 (0.885-0.925)	0.869	0.801 (0.767-0.831)	0.876 (0.846-0.901)	0.837	0.780 (0.735-0.818)	0.911 (0.875-0.937)	0.840
Consolidation	0.773 (0.720-0.819)	0.776 (0.723-0.821)	0.774	0.659 (0.606-0.708)	0.791 (0.739-0.835)	0.719	0.666 (0.634-0.697)	0.802 (0.771-0.830)	0.728	0.660 (0.609-0.708)	0.769 (0.718-0.813)	0.710	0.714 (0.643-0.776)	0.735 (0.664-0.796)	0.725
Effusion	0.636 (0.575-0.693)	0.904 (0.852-0.940)	0.747	0.663 (0.597-0.724)	0.868 (0.807-0.912)	0.752	0.624 (0.573-0.672)	0.751 (0.699-0.796)	0.682	0.632 (0.572-0.688)	0.760 (0.699-0.812)	0.690	0.773 (0.729-0.812)	0.882 (0.844-0.912)	0.824
Emphysema	0.808 (0.760-0.848)	0.790 (0.741-0.831)	0.799	0.715 (0.654-0.769)	0.796 (0.737-0.845)	0.753	0.523 (0.485-0.560)	0.820 (0.781-0.853)	0.638	0.492 (0.452-0.533)	0.915 (0.879-0.941)	0.640	0.573 (0.502-0.641)	0.529 (0.461-0.596)	0.550
Fibrosis	0.769 (0.705-0.823)	0.735 (0.671-0.791)	0.752	0.631 (0.564-0.693)	0.767 (0.699-0.823)	0.692	0.756 (0.714-0.794)	0.647 (0.605-0.687)	0.697	0.729 (0.646-0.798)	0.595 (0.517-0.668)	0.655	0.812 (0.736-0.871)	0.698 (0.620-0.766)	0.751
Fracture	0.853 (0.784-0.903)	0.795 (0.722-0.852)	0.823	0.845 (0.778-0.894)	0.828 (0.760-0.880)	0.836	0.764 (0.710-0.811)	0.836 (0.784-0.877)	0.798	0.842 (0.796-0.879)	0.775 (0.726-0.818)	0.807	0.847 (0.756-0.908)	0.692 (0.598-0.773)	0.762
Mass	0.688 (0.444-0.858)	0.611 (0.386-0.797)	0.647	0.542 (0.351-0.721)	0.591 (0.387-0.767)	0.565	0.678 (0.551-0.783)	0.678 (0.551-0.783)	0.678	0.537 (0.387-0.679)	0.846 (0.665-0.939)	0.657	0.818 (0.615-0.927)	0.783 (0.581-0.903)	0.800
Nodule	0.659 (0.627-0.691)	0.676 (0.643-0.707)	0.667	0.574 (0.543-0.604)	0.562 (0.531-0.592)	0.568	0.608 (0.587-0.628)	0.714 (0.693-0.734)	0.657	0.538 (0.514-0.563)	0.650 (0.624-0.675)	0.589	0.565 (0.510-0.619)	0.700 (0.640-0.753)	0.625
Pleural thickening	0.633 (0.523-0.731)	0.714 (0.599-0.807)	0.671	0.711 (0.614-0.792)	0.711 (0.614-0.792)	0.711	0.709 (0.634-0.774)	0.679 (0.604-0.745)	0.693	0.700 (0.592-0.789)	0.622 (0.519-0.715)	0.659	0.758 (0.638-0.848)	0.495 (0.396-0.594)	0.599
Pneumatosis	0.625 (0.386-0.815)	0.500 (0.299-0.701)	0.556	0.417 (0.193-0.680)	0.417 (0.193-0.680)	0.417	0.571 (0.326-0.786)	0.444 (0.246-0.663)	0.500	0.435 (0.256-0.632)	0.417 (0.245-0.612)	0.426	0.835 (0.751-0.894)	0.717 (0.630-0.790)	0.771
Pneumothorax	1.000 (0.785-1.000)	0.824 (0.590-0.938)	0.903	0.826 (0.629-0.930)	0.792 (0.595-0.908)	0.809	0.821 (0.673-0.910)	0.640 (0.501-0.759)	0.719	0.700 (0.481-0.855)	0.700 (0.481-0.855)	0.700	0.928 (0.841-0.969)	0.821 (0.721-0.890)	0.871
Postoperative Metal	0.869 (0.788-0.922)	0.905 (0.830-0.949)	0.887	0.933 (0.787-0.982)	0.966 (0.828-0.994)	0.949	0.933 (0.702-0.988)	0.933 (0.702-0.988)	0.933	1.000 (0.918-1.000)	0.878 (0.758-0.943)	0.935	0.924 (0.877-0.954)	0.977 (0.943-0.991)	0.950
Venipuncture	0.900 (0.596-0.982)	0.600 (0.357-0.802)	0.720	1.000 (0.806-1.000)	0.842 (0.624-0.945)	0.914	0.850 (0.640-0.948)	1.000 (0.816-1.000)	0.919	0.750 (0.301-0.954)	1.000 (0.439-1.000)	0.857	0.973 (0.906-0.992)	0.899 (0.813-0.948)	0.934
Mean	0.751	0.74	0.738	0.700	0.755	0.720	0.712	0.748	0.724	0.673	0.736	0.693	0.783	0.727	0.746

The 95% Wilson confidence interval was applied to both the precision and recall of different abnormality on each test dataset. IoU-T: intersection over union threshold.

(Source data are provided as a Source Data file.)

Supplementary Table 9 Average IoU value of each abnormality achieved by the YOLOX model on the external test dataset (a total of 10,945 CXRs from four multicentres).

Abnormality （category）	Average IoU (mean±SD)
Atelectasis	0.590±0.253
Calcification	0.762±0.175
Consolidation	0.714±0.195
Effusion	0.550±0.146
Emphysema	0.917±0.037
Fibrosis	0.686±0.196
Fracture	0.769±0.146
Mass	0.792±0.166
Nodule	0.658±0.212
Pleural thickening	0.661±0.174
Pneumatosis	0.458±0.271
Pneumothorax	0.734±0.175
Postoperative metal	0.841±0.154
Venipuncture	0.800±0.111

IoU: intersection over union.

(Source data are provided as a Source Data file.)

Supplementary Table 10 Performance comparison among the YOLOX model, the best junior radiologist and the best senior radiologist on the held-out test dataset with an IoU-T of 0.5.

	YOLOX			Junior 3 (Best)					Senior 2 (Best)				
	**Precision**	**Recall**	**F1**	**Precision**	***P*-value**	**Recall**	***P*-value**	**F1**	**Precision**	***P*-value**	**Recall**	***P*-value**	**F1**
**Atelectasis**	0.455 (0.213-0.720)	0.208 (0.092-0.405)	0.286	0.600 (0.387-0.781)	0.436	0.500 (0.314-0.686)	0.039	0.545	0.486 (0.334-0.641)	0.852	0.750 (0.551-0.880)	0.002	0.590
**Calcification**	0.755 (0.730-0.778)	0.732 (0.707-0.755)	0.743	0.416 (0.388-0.444)	0.000	0.385 (0.359-0.412)	0.000	0.400	0.523 (0.494-0.552)	0.000	0.469 (0.441-0.496)	0.000	0.495
**Consolidation**	0.650 (0.615-0.684)	0.611 (0.576-0.644)	0.630	0.370 (0.339-0.402)	0.000	0.429 (0.395-0.465)	0.000	0.398	0.556 (0.516-0.595)	0.000	0.431 (0.396-0.466)	0.000	0.485
**Effusion**	0.707 (0.683-0.730)	0.683 (0.659-0.706)	0.695	0.336 (0.309-0.365)	0.000	0.242 (0.221-0.264)	0.000	0.281	0.381 (0.354-0.410)	0.000	0.297 (0.274-0.320)	0.000	0.334
**Emphysema**	0.594 (0.560-0.628)	0.707 (0.671-0.740)	0.646	0.887 (0.850-0.916)	0.000	0.450 (0.413-0.488)	0.000	0.597	0.796 (0.762-0.826)	0.000	0.698 (0.662-0.731)	0.757	0.744
**Fibrosis**	0.629 (0.592-0.665)	0.623 (0.585-0.659)	0.626	0.322 (0.287-0.358)	0.000	0.328 (0.293-0.364)	0.000	0.325	0.589 (0.537-0.640)	0.214	0.308 (0.274-0.344)	0.000	0.405
**Fracture**	0.783 (0.746-0.815)	0.790 (0.753-0.822)	0.786	0.654 (0.607-0.698)	0.000	0.510 (0.468-0.553)	0.000	0.573	0.706 (0.658-0.750)	0.009	0.492 (0.449-0.534)	0.000	0.580
**Mass**	0.682 (0.577-0.772)	0.744 (0.637-0.827)	0.712	0.659 (0.555-0.750)	0.745	0.744 (0.637-0.827)	1.000	0.699	0.881 (0.782-0.938)	0.004	0.756 (0.651-0.838)	1.000	0.814
**Nodule**	0.396 (0.375-0.417)	0.534 (0.509-0.559)	0.455	0.221 (0.198-0.246)	0.000	0.169 (0.151-0.188)	0.000	0.192	0.319 (0.291-0.347)	0.000	0.223 (0.202-0.244)	0.000	0.262
**Pleural thickening**	0.610 (0.536-0.680)	0.398 (0.341-0.458)	0.482	0.227 (0.188-0.271)	0.000	0.333 (0.279-0.392)	0.115	0.270	0.303 (0.249-0.364)	0.000	0.280 (0.230-0.337)	0.007	0.291
**Pneumatosis**	0.677 (0.613-0.735)	0.602 (0.540-0.660)	0.637	0.486 (0.426-0.547)	0.000	0.494 (0.433-0.555)	0.082	0.490	0.611 (0.542-0.676)	0.157	0.482 (0.421-0.544)	0.025	0.539
**Pneumothorax**	0.874 (0.818-0.914)	0.743 (0.681-0.797)	0.803	0.744 (0.682-0.798)	0.001	0.748 (0.685-0.801)	0.888	0.746	0.732 (0.664-0.790)	0.001	0.650 (0.583-0.710)	0.027	0.688
**Postoperative Metal**	0.952 (0.932-0.967)	0.850 (0.820-0.875)	0.898	0.945 (0.924-0.960)	0.574	0.891 (0.864-0.912)	0.017	0.917	0.965 (0.947-0.976)	0.287	0.909 (0.884-0.929)	0.000	0.936
**Venipuncture**	0.934 (0.906-0.955)	0.889 (0.856-0.915)	0.911	0.958 (0.935-0.974)	0.121	0.958 (0.935-0.974)	0.000	0.958	0.921 (0.892-0.943)	0.460	0.919 (0.890-0.941)	0.117	0.920
**Mean**	0.693	0.651	0.665	0.559	-	0.513	-	0.528	0.626	-	0.547	-	0.577

The precision of different abnormality between YOLOX models and radiologist (Junior or Senior) were performed with chi-square test (two-sided).

The recall of different abnormality between YOLOX models and radiologist (Junior or Senior) were performed with related-samples McNemar test (two-sided).

The 95% Wilson confidence interval was applied to both the precision and recall of different abnormality.

IoU-T: intersection over union threshold.

(Source data are provided as a Source Data file.)

Supplementary Table 11 Performance of the other four radiologists on the held-out test dataset with an IoU-T of 0.5.

	Junior 1			Junior 2			Senior 1			Senior 3		
	**Precision**	**Recall**	**F1**	**Precision**	**Recall**	**F1**	**Precision**	**Recall**	**F1**	**Precision**	**Recall**	**F1**
**Atelectasis**	0.727 (0.434-0.903)	0.333 (0.180-0.533)	0.457	0.750 (0.505-0.898)	0.500 (0.314-0.686)	0.600	0.636 (0.430-0.803)	0.583 (0.388-0.755)	0.609	0.432 (0.287-0.591)	0.667 (0.467-0.820)	0.525
**Calcification**	0.388 (0.361-0.417)	0.351 (0.325-0.378)	0.369	0.459 (0.430-0.487)	0.428 (0.401-0.455)	0.443	0.515 (0.485-0.546)	0.416 (0.389-0.443)	0.460	0.545 (0.517-0.573)	0.522 (0.495-0.550)	0.534
**Consolidation**	0.439 (0.408-0.471)	0.536 (0.500-0.570)	0.483	0.280 (0.256-0.304)	0.493 (0.458-0.528)	0.357	0.445 (0.410-0.481)	0.433 (0.399-0.469)	0.439	0.601 (0.555-0.645)	0.347 (0.314-0.381)	0.440
**Effusion**	0.222 (0.199-0.246)	0.177 (0.159-0.197)	0.197	0.407 (0.382-0.433)	0.386 (0.362-0.411)	0.396	0.312 (0.286-0.339)	0.244 (0.223-0.266)	0.273	0.236 (0.211-0.262)	0.170 (0.152-0.190)	0.198
**Emphysema**	0.802 (0.766-0.834)	0.617 (0.580-0.653)	0.698	0.866 (0.835-0.892)	0.701 (0.665-0.734)	0.775	0.783 (0.752-0.812)	0.817 (0.786-0.844)	0.800	0.830 (0.794-0.860)	0.607 (0.570-0.643)	0.701
**Fibrosis**	0.280 (0.250-0.312)	0.344 (0.309-0.381)	0.309	0.594 (0.525-0.659)	0.180 (0.153-0.211)	0.277	0.405 (0.363-0.448)	0.313 (0.279-0.349)	0.353	0.491 (0.438-0.544)	0.250 (0.218-0.284)	0.331
**Fracture**	0.660 (0.598-0.717)	0.298 (0.261-0.338)	0.411	0.762 (0.700-0.814)	0.306 (0.268-0.346)	0.436	0.623 (0.567-0.675)	0.356 (0.317-0.398)	0.453	0.715 (0.667-0.759)	0.490 (0.447-0.532)	0.581
**Mass**	0.546 (0.452-0.637)	0.756 (0.651-0.838)	0.634	0.837 (0.710-0.915)	0.526 (0.416-0.633)	0.646	0.662 (0.549-0.760)	0.628 (0.517-0.727)	0.645	0.945 (0.851-0.981)	0.667 (0.556-0.761)	0.782
**Nodule**	0.486 (0.460-0.512)	0.451 (0.426-0.476)	0.468	0.562 (0.520-0.603)	0.205 (0.185-0.226)	0.300	0.320 (0.297-0.344)	0.306 (0.283-0.329)	0.313	0.273 (0.246-0.302)	0.174 (0.156-0.194)	0.213
**Pleural thickening**	0.328 (0.265-0.398)	0.231 (0.184-0.286)	0.271	0.463 (0.360-0.571)	0.144 (0.107-0.191)	0.220	0.252 (0.207-0.302)	0.303 (0.251-0.361)	0.275	0.461 (0.396-0.527)	0.379 (0.322-0.439)	0.416
**Pneumatosis**	0.340 (0.279-0.406)	0.287 (0.234-0.346)	0.311	0.423 (0.357-0.492)	0.339 (0.283-0.399)	0.376	0.536 (0.466-0.606)	0.410 (0.351-0.472)	0.465	0.480 (0.407-0.554)	0.335 (0.279-0.395)	0.394
**Pneumothorax**	0.661 (0.595-0.720)	0.673 (0.607-0.732)	0.667	0.849 (0.782-0.898)	0.579 (0.512-0.644)	0.689	0.670 (0.600-0.733)	0.589 (0.522-0.653)	0.627	0.752 (0.690-0.805)	0.752 (0.690-0.805)	0.752
**Postoperative Metal**	0.941 (0.920-0.957)	0.877 (0.850-0.900)	0.908	0.954 (0.934-0.968)	0.881 (0.855-0.904)	0.916	0.953 (0.932-0.968)	0.805 (0.773-0.834)	0.873	0.982 (0.969-0.990)	0.926 (0.903-0.943)	0.953
**Venipuncture**	0.923 (0.894-0.945)	0.915 (0.884-0.937)	0.919	0.940 (0.914-0.959)	0.912 (0.882-0.935)	0.926	0.926 (0.897-0.947)	0.921 (0.892-0.943)	0.924	0.909 (0.878-0.932)	0.921 (0.892-0.943)	0.915
**Mean**	0.553	0.489	0.507	0.653	0.470	0.525	0.574	0.509	0.536	0.618	0.515	0.552

The 95% Wilson confidence interval was applied to both the precision and recall of different abnormality. IoU-T: intersection over union threshold.

(Source data are provided as a Source Data file.)

Supplementary Table 12 Performance comparison among the YOLOX model, the best junior radiologist and the best senior radiologist on the held-out test dataset with an IoU-T of 0.3.

	YOLOX			Junior 3 (Best)					Senior 2 (Best)				
	Precision	Recall	F1	Precision	*P*-value	Recall	*P*-value	F1	Precision	*P*-value	Recall	*P*-value	F1
**Atelectasis**	0.350 (0.181-0.567)	0.292 (0.149-0.492)	0.318	0.650 (0.433-0.819)	0.058	0.542 (0.351-0.721)	0.109	0.591	0.486 (0.334-0.641)	0.322	0.750 (0.551-0.880)	0.013	0.590
**Calcification**	0.841 (0.820-0.861)	0.815 (0.793-0.836)	0.828	0.691 (0.664-0.717)	0.000	0.640 (0.613-0.666)	0.000	0.664	0.728 (0.701-0.753)	0.000	0.652 (0.625-0.678)	0.000	0.688
**Consolidation**	0.684 (0.651-0.714)	0.730 (0.697-0.760)	0.706	0.536 (0.504-0.569)	0.000	0.622 (0.588-0.656)	0.000	0.576	0.758 (0.722-0.791)	0.002	0.587 (0.552-0.622)	0.000	0.662
**Effusion**	0.819 (0.798-0.838)	0.797 (0.776-0.817)	0.808	0.654 (0.626-0.682)	0.000	0.470 (0.445-0.495)	0.000	0.547	0.699 (0.672-0.724)	0.000	0.544 (0.519-0.569)	0.000	0.612
**Emphysema**	0.594 (0.560-0.628)	0.707 (0.671-0.740)	0.646	0.887 (0.850-0.916)	0.000	0.450 (0.413-0.488)	0.000	0.597	0.796 (0.762-0.826)	0.000	0.698 (0.662-0.731)	0.757	0.744
**Fibrosis**	0.684 (0.647-0.718)	0.677 (0.640-0.711)	0.680	0.519 (0.482-0.557)	0.000	0.529 (0.491-0.567)	0.000	0.524	0.802 (0.757-0.840)	0.000	0.420 (0.383-0.457)	0.000	0.551
**Fracture**	0.816 (0.781-0.846)	0.824 (0.789-0.854)	0.820	0.767 (0.724-0.805)	0.062	0.598 (0.556-0.639)	0.000	0.672	0.795 (0.751-0.833)	0.434	0.553 (0.511-0.595)	0.000	0.653
**Mass**	0.660 (0.559-0.747)	0.795 (0.692-0.870)	0.721	0.693 (0.590-0.780)	0.628	0.782 (0.678-0.859)	1.000	0.735	0.925 (0.837-0.968)	0.000	0.795 (0.692-0.870)	1.000	0.855
**Nodule**	0.560 (0.537-0.582)	0.679 (0.655-0.702)	0.614	0.395 (0.367-0.423)	0.000	0.301 (0.279-0.325)	0.000	0.341	0.509 (0.479-0.539)	0.008	0.355 (0.332-0.380)	0.000	0.418
**Pleural thickening**	0.547 (0.486-0.606)	0.534 (0.474-0.593)	0.540	0.325 (0.280-0.373)	0.000	0.477 (0.418-0.537)	0.115	0.387	0.410 (0.350-0.472)	0.002	0.379 (0.322-0.439)	0.000	0.394
**Pneumatosis**	0.807 (0.750-0.854)	0.717 (0.658-0.769)	0.759	0.647 (0.587-0.703)	0.000	0.657 (0.597-0.713)	0.704	0.652	0.783 (0.720-0.835)	0.536	0.618 (0.556-0.675)	0.253	0.690
**Pneumothorax**	0.911 (0.863-0.944)	0.818 (0.761-0.864)	0.862	0.819 (0.762-0.864)	0.007	0.822 (0.766-0.868)	0.878	0.821	0.837 (0.778-0.883)	0.028	0.743 (0.681-0.797)	0.072	0.787
**Postoperative Metal**	0.973 (0.956-0.983)	0.868 (0.840-0.892)	0.917	0.998 (0.991-1.000)	0.000	0.941 (0.920-0.956)	0.000	0.969	0.992 (0.981-0.997)	0.011	0.935 (0.913-0.951)	0.000	0.962
**Venipuncture**	0.961 (0.938-0.976)	0.915 (0.884-0.937)	0.937	0.984 (0.967-0.992)	0.043	0.984 (0.967-0.992)	0.000	0.984	0.975 (0.955-0.986)	0.270	0.972 (0.952-0.984)	0.000	0.973
**Mean**	0.729	0.726	0.725	0.683	-	0.630	-	0.647	0.750	-	0.643	-	0.684

The precision of different abnormality between YOLOX models and radiologist (Junior or Senior) were performed with chi-square test (two-sided).

The recall of different abnormality between YOLOX models and radiologist (Junior or Senior) were performed with related-samples McNemar test (two-sided).

The 95% Wilson confidence interval was applied to both the precision and recall of different abnormality.

IoU-T: intersection over union threshold.

(Source data are provided as a Source Data file.)

Supplementary Table 13 Performance of the other four radiologists on the held-out test dataset with an IoU-T of 0.3.

	Junior 1			Junior 2			Senior 1			Senior 3		
	Precision	Recall	F1	Precision	Recall	F1	Precision	Recall	F1	Precision	Recall	F1
**Atelectasis**	0.909 (0.623-0.984)	0.417 (0.245-0.612)	0.571	0.875 (0.640-0.965)	0.583 (0.388-0.755)	0.700	0.773 (0.566-0.899)	0.708 (0.508-0.851)	0.739	0.486 (0.334-0.641)	0.750 (0.551-0.880)	0.590
**Calcification**	0.546 (0.517-0.575)	0.495 (0.467-0.522)	0.519	0.673 (0.646-0.700)	0.629 (0.602-0.655)	0.651	0.753 (0.725-0.778)	0.608 (0.581-0.634)	0.672	0.754 (0.729-0.777)	0.722 (0.697-0.746)	0.738
**Consolidation**	0.562 (0.530-0.593)	0.686 (0.652-0.717)	0.618	0.413 (0.387-0.439)	0.728 (0.696-0.759)	0.527	0.614 (0.578-0.648)	0.598 (0.563-0.632)	0.606	0.798 (0.758-0.833)	0.461 (0.426-0.496)	0.584
**Effusion**	0.565 (0.537-0.593)	0.452 (0.427-0.477)	0.502	0.700 (0.675-0.723)	0.663 (0.639-0.687)	0.681	0.654 (0.627-0.681)	0.512 (0.487-0.537)	0.575	0.647 (0.618-0.675)	0.467 (0.442-0.492)	0.542
**Emphysema**	0.802 (0.766-0.834)	0.618 (0.581-0.654)	0.698	0.866 (0.835-0.892)	0.701 (0.665-0.734)	0.775	0.783 (0.752-0.812)	0.817 (0.786-0.844)	0.800	0.830 (0.794-0.860)	0.607 (0.570-0.643)	0.701
**Fibrosis**	0.412 (0.379-0.446)	0.507 (0.469-0.545)	0.454	0.847 (0.790-0.890)	0.257 (0.225-0.292)	0.394	0.599 (0.556-0.641)	0.463 (0.426-0.501)	0.522	0.734 (0.684-0.778)	0.373 (0.337-0.410)	0.495
**Fracture**	0.842 (0.791-0.883)	0.381 (0.341-0.423)	0.525	0.953 (0.916-0.974)	0.383 (0.342-0.425)	0.546	0.767 (0.717-0.811)	0.439 (0.397-0.481)	0.558	0.814 (0.771-0.850)	0.557 (0.515-0.599)	0.661
**Mass**	0.593 (0.498-0.681)	0.821 (0.721-0.890)	0.688	0.837 (0.710-0.915)	0.526 (0.416-0.633)	0.646	0.716 (0.605-0.806)	0.679 (0.570-0.773)	0.697	0.982 (0.904-0.997)	0.692 (0.583-0.784)	0.812
**Nodule**	0.580 (0.554-0.605)	0.538 (0.513-0.563)	0.558	0.736 (0.698-0.771)	0.268 (0.247-0.291)	0.393	0.479 (0.454-0.505)	0.457 (0.433-0.483)	0.468	0.480 (0.449-0.512)	0.306 (0.284-0.330)	0.374
**Pleural thickening**	0.505 (0.434-0.576)	0.356 (0.301-0.416)	0.418	0.720 (0.614-0.805)	0.223 (0.177-0.277)	0.341	0.396 (0.344-0.451)	0.477 (0.418-0.537)	0.433	0.590 (0.523-0.653)	0.485 (0.425-0.545)	0.532
**Pneumatosis**	0.566 (0.499-0.631)	0.478 (0.417-0.540)	0.518	0.667 (0.599-0.728)	0.534 (0.472-0.595)	0.593	0.740 (0.673-0.797)	0.566 (0.504-0.626)	0.641	0.743 (0.673-0.802)	0.518 (0.456-0.579)	0.610
**Pneumothorax**	0.766 (0.706-0.817)	0.780 (0.720-0.831)	0.773	0.911 (0.854-0.947)	0.621 (0.555-0.684)	0.739	0.856 (0.799-0.899)	0.752 (0.690-0.805)	0.801	0.855 (0.802-0.896)	0.855 (0.802-0.896)	0.855
**Postoperative Metal**	0.982 (0.968-0.990)	0.915 (0.891-0.934)	0.947	0.995 (0.986-0.998)	0.919 (0.896-0.938)	0.956	0.989 (0.977-0.995)	0.836 (0.806-0.862)	0.906	0.995 (0.986-0.998)	0.938 (0.917-0.954)	0.966
**Venipuncture**	0.986 (0.970-0.994)	0.977 (0.958-0.987)	0.981	0.983 (0.966-0.992)	0.954 (0.930-0.970)	0.968	0.970 (0.949-0.982)	0.965 (0.944-0.979)	0.968	0.970 (0.950-0.983)	0.984 (0.967-0.992)	0.977
**Mean**	0.687	0.601	0.627	0.798	0.571	0.636	0.721	0.634	0.670	0.763	0.622	0.674

The 95% Wilson confidence interval was applied to both the precision and recall of different abnormality. IoU-T: intersection over union threshold.

(Source data are provided as a Source Data file.)

Supplementary Table 14 Performance comparison among the YOLOX model, the best junior radiologist and the best senior radiologist on the held-out test dataset with an IoU-T of 0.1.

	YOLOX			Junior 3 (Best)					Senior 2 (Best)				
	**Precision**	**Recall**	**F1**	**Precision**	***P*-value**	**Recall**	***P*-value**	**F1**	**Precision**	***P*-value**	**Recall**	***P*-value**	**F1**
**Atelectasis**	0.350 (0.181-0.567)	0.292 (0.149-0.492)	0.318	0.650 (0.433-0.819)	0.058	0.542 (0.351-0.721)	0.109	0.591	0.486 (0.334-0.641)	0.322	0.750 (0.551-0.880)	0.013	0.590
**Calcification**	0.881 (0.862-0.898)	0.854 (0.833-0.872)	0.867	0.819 (0.796-0.840)	0.000	0.759 (0.735-0.782)	0.000	0.788	0.856 (0.834-0.875)	0.066	0.766 (0.742-0.789)	0.000	0.808
**Consolidation**	0.708 (0.676-0.738)	0.755 (0.724-0.784)	0.731	0.609 (0.576-0.640)	0.000	0.706 (0.673-0.737)	0.017	0.654	0.816 (0.783-0.845)	0.000	0.633 (0.598-0.666)	0.000	0.713
**Effusion**	0.831 (0.813-0.849)	0.901 (0.885-0.915)	0.865	0.911 (0.893-0.927)	0.000	0.655 (0.630-0.678)	0.000	0.762	0.869 (0.848-0.887)	0.007	0.676 (0.652-0.699)	0.000	0.760
**Emphysema**	0.594 (0.560-0.628)	0.707 (0.671-0.740)	0.646	0.887 (0.850-0.916)	0.000	0.450 (0.413-0.488)	0.000	0.597	0.796 (0.762-0.826)	0.000	0.698 (0.662-0.731)	0.757	0.744
**Fibrosis**	0.694 (0.658-0.728)	0.692 (0.656-0.726)	0.693	0.602 (0.564-0.638)	0.000	0.614 (0.576-0.650)	0.001	0.608	0.848 (0.806-0.882)	0.000	0.444 (0.406-0.482)	0.000	0.582
**Fracture**	0.816 (0.781-0.846)	0.824 (0.789-0.854)	0.820	0.788 (0.747-0.825)	0.288	0.615 (0.573-0.656)	0.000	0.691	0.827 (0.786-0.863)	0.657	0.576 (0.534-0.617)	0.000	0.679
**Mass**	0.681 (0.581-0.766)	0.821 (0.721-0.890)	0.744	0.705 (0.602-0.790)	0.729	0.795 (0.692-0.870)	0.824	0.747	0.940 (0.856-0.977)	0.000	0.808 (0.707-0.880)	1.000	0.869
**Nodule**	0.614 (0.591-0.636)	0.709 (0.685-0.731)	0.658	0.622 (0.594-0.649)	0.655	0.474 (0.450-0.500)	0.000	0.538	0.726 (0.699-0.752)	0.000	0.507 (0.482-0.532)	0.000	0.597
**Pleural thickening**	0.626 (0.559-0.688)	0.500 (0.440-0.560)	0.556	0.374 (0.327-0.423)	0.000	0.549 (0.489-0.608)	0.440	0.445	0.475 (0.414-0.538)	0.001	0.439 (0.381-0.500)	0.228	0.457
**Pneumatosis**	0.810 (0.757-0.854)	0.817 (0.764-0.860)	0.813	0.725 (0.668-0.777)	0.024	0.737 (0.679-0.788)	0.229	0.731	0.848 (0.792-0.892)	0.287	0.669 (0.609-0.725)	0.007	0.748
**Pneumothorax**	0.932 (0.888-0.960)	0.836 (0.781-0.880)	0.882	0.847 (0.792-0.889)	0.006	0.850 (0.797-0.892)	0.755	0.848	0.884 (0.831-0.922)	0.103	0.785 (0.725-0.835)	0.177	0.832
**Postoperative Metal**	0.976 (0.960-0.986)	0.871 (0.843-0.894)	0.920	1.000 (0.994-1.000)	0.000	0.942 (0.922-0.958)	0.000	0.970	1.000 (0.994-1.000)	0.000	0.942 (0.922-0.958)	0.000	0.970
**Venipuncture**	0.960 (0.937-0.975)	0.938 (0.911-0.957)	0.949	0.984 (0.967-0.992)	0.033	0.984 (0.967-0.992)	0.000	0.984	0.975 (0.955-0.986)	0.226	0.972 (0.952-0.984)	0.001	0.973
**Mean**	0.748	0.751	0.747	0.752	-	0.691	-	0.711	0.811	-	0.690	-	0.737

The precision of different abnormality between YOLOX models and radiologist (Junior or Senior) were performed with chi-square test (two-sided).

The recall of different abnormality between YOLOX models and radiologist (Junior or Senior) were performed with related-samples McNemar test (two-sided).

The 95% Wilson confidence interval was applied to both the precision and recall of different abnormality.

IoU-T: intersection over union threshold.

(Source data are provided as a Source Data file.)

Supplementary Table 15 Performance of the other four radiologists with on the held-out test dataset with an IoU-T of 0.1.

	Junior 1			Junior 2			Senior 1			Senior 3		
	**Precision**	**Recall**	**F1**	**Precision**	**Recall**	**F1**	**Precision**	**Recall**	**F1**	**Precision**	**Recall**	**F1**
**Atelectasis**	1.000 (0.741-1.000)	0.458 (0.279-0.649)	0.629	0.875 (0.640-0.965)	0.583 (0.388-0.755)	0.700	0.773 (0.566-0.899)	0.708 (0.508-0.851)	0.739	0.486 (0.334-0.641)	0.750 (0.551-0.880)	0.590
**Calcification**	0.810 (0.786-0.831)	0.734 (0.709-0.757)	0.770	0.787 (0.763-0.809)	0.735 (0.710-0.759)	0.760	0.902 (0.883-0.919)	0.728 (0.703-0.752)	0.806	0.857 (0.837-0.876)	0.822 (0.800-0.842)	0.839
**Consolidation**	0.609 (0.577-0.639)	0.743 (0.711-0.772)	0.669	0.469 (0.442-0.495)	0.827 (0.798-0.852)	0.598	0.684 (0.650-0.716)	0.666 (0.632-0.699)	0.675	0.854 (0.818-0.884)	0.493 (0.458-0.528)	0.625
**Effusion**	0.838 (0.816-0.858)	0.670 (0.646-0.693)	0.745	0.842 (0.823-0.860)	0.799 (0.778-0.818)	0.820	0.851 (0.830-0.871)	0.666 (0.642-0.689)	0.747	0.900 (0.880-0.916)	0.649 (0.624-0.672)	0.754
**Emphysema**	0.802 (0.766-0.834)	0.618 (0.581-0.654)	0.698	0.866 (0.835-0.892)	0.701 (0.665-0.734)	0.775	0.783 (0.752-0.812)	0.817 (0.786-0.844)	0.800	0.830 (0.794-0.860)	0.607 (0.570-0.643)	0.701
**Fibrosis**	0.487 (0.452-0.521)	0.598 (0.561-0.635)	0.537	0.886 (0.835-0.923)	0.269 (0.237-0.304)	0.413	0.689 (0.647-0.727)	0.532 (0.494-0.570)	0.601	0.802 (0.756-0.841)	0.408 (0.371-0.445)	0.540
**Fracture**	0.892 (0.847-0.925)	0.403 (0.363-0.446)	0.556	0.972 (0.940-0.987)	0.390 (0.350-0.432)	0.557	0.797 (0.748-0.838)	0.456 (0.414-0.498)	0.580	0.825 (0.782-0.860)	0.565 (0.522-0.606)	0.670
**Mass**	0.611 (0.517-0.698)	0.846 (0.750-0.910)	0.710	0.837 (0.710-0.915)	0.526 (0.416-0.633)	0.646	0.730 (0.619-0.818)	0.692 (0.583-0.784)	0.711	1.000 (0.935-1.000)	0.705 (0.596-0.795)	0.827
**Nodule**	0.591 (0.565-0.616)	0.548 (0.523-0.573)	0.569	0.853 (0.821-0.880)	0.311 (0.288-0.335)	0.456	0.569 (0.543-0.594)	0.543 (0.518-0.568)	0.556	0.666 (0.636-0.695)	0.425 (0.400-0.450)	0.519
**Pleural thickening**	0.554 (0.482-0.623)	0.390 (0.333-0.450)	0.458	0.793 (0.693-0.866)	0.246 (0.198-0.302)	0.376	0.453 (0.399-0.508)	0.545 (0.485-0.604)	0.495	0.664 (0.598-0.723)	0.545 (0.485-0.604)	0.599
**Pneumatosis**	0.731 (0.668-0.786)	0.618 (0.556-0.675)	0.670	0.771 (0.708-0.824)	0.618 (0.556-0.675)	0.686	0.885 (0.833-0.923)	0.677 (0.617-0.732)	0.767	0.880 (0.824-0.920)	0.614 (0.552-0.672)	0.723
**Pneumothorax**	0.830 (0.775-0.874)	0.846 (0.791-0.888)	0.838	0.932 (0.879-0.962)	0.636 (0.569-0.697)	0.756	0.894 (0.841-0.930)	0.785 (0.725-0.835)	0.836	0.887 (0.838-0.923)	0.883 (0.833-0.920)	0.885
**Postoperative Metal**	1.000 (0.994-1.000)	0.932 (0.910-0.949)	0.965	1.000 (0.994-1.000)	0.924 (0.901-0.942)	0.961	1.000 (0.993-1.000)	0.845 (0.815-0.871)	0.916	1.000 (0.994-1.000)	0.942 (0.922-0.958)	0.970
**Venipuncture**	0.986 (0.970-0.994)	0.977 (0.958-0.987)	0.981	0.988 (0.972-0.995)	0.958 (0.935-0.974)	0.973	0.974 (0.955-0.986)	0.970 (0.949-0.982)	0.972	0.970 (0.950-0.983)	0.984 (0.967-0.992)	0.977
**Mean**	0.767	0.670	0.699	0.848	0.609	0.677	0.785	0.688	0.729	0.830	0.671	0.730

The 95% Wilson confidence interval was applied to both the precision and recall of different abnormality. IoU-T: intersection over union threshold.

(Source data are provided as a Source Data file.)

Supplementary Table 16 ANOVA for F1-score achieved by the YOLOX model, junior group and senior group on the held-out test dataset with an IoU-T of 0.5.

	Sum of Squares	Df	Mean Square	F	*P*
**Between Groups**	0.220	2	0.110	2.319	0.104
**Within Groups**	4.507	95	0.047		
**Total**	4.727	97			

Comparisons were performed with a two-sided Analysis of Variance (ANOVA).

Df: Degree of freedom. IoU-T: intersection over union threshold.

(Source data are provided as a Source Data file.)

Supplementary Table 17 Multiple comparisons among the YOLOX model, junior group and senior group on the held-out test dataset with an IoU-T of 0.5.

		Mean Difference			95% Confidence Interval	
**(I) Group**	**(J) Group**	**(I-J)**	**Std. Error**	***P***	**Lower Bound**	**Upper Bound**
**Junior group**	**Senior group**	-0.035	0.048	0.461	-0.130	0.059
**Senior group**	**YOLOX**	-0.110	0.067	0.106	-0.243	0.024
**YOLOX**	**Junior group**	0.145*	0.067	0.034	0.011	0.278

*. The mean difference is significant at the 0.05 level. Multiple comparisons were performed with a two-sided LSD method.

(Source data are provided as a Source Data file.)

Supplementary Table 18 Performance comparison among the YOLOX model, the best junior radiologist and the best senior radiologist on the recombination test dataset with an IoU-T of 0.5.

	YOLOX			Junior 1 (Best)					Senior 2 (Best)				
	**Precision**	**Recall**	**F1**	**Precision**	***P*-value**	**Recall**	***P*-value**	**F1**	**Precision**	***P*-value**	**Recall**	***P*-value**	**F1**
**Atelectasis**	0.471 (0.262-0.690)	0.615 (0.355-0.823)	0.533	0.667 (0.354-0.879)	0.340	0.462 (0.232-0.709)	1.000	0.545	0.700 (0.397-0.892)	0.247	0.538 (0.291-0.768)	1.000	0.609
**Calcification**	0.801 (0.766-0.832)	0.746 (0.709-0.779)	0.772	0.668 (0.624-0.709)	0.000	0.518 (0.478-0.558)	0.000	0.584	0.672 (0.633-0.709)	0.000	0.659 (0.620-0.696)	0.000	0.666
**Consolidation**	0.569 (0.526-0.612)	0.671 (0.625-0.713)	0.616	0.544 (0.499-0.588)	0.417	0.610 (0.563-0.655)	0.064	0.575	0.624 (0.573-0.673)	0.108	0.516 (0.469-0.563)	0.001	0.565
**Effusion**	0.389 (0.336-0.445)	0.490 (0.427-0.552)	0.434	0.319 (0.262-0.381)	0.092	0.307 (0.252-0.368)	0.000	0.313	0.419 (0.353-0.487)	0.510	0.353 (0.295-0.415)	0.002	0.383
**Emphysema**	0.660 (0.612-0.705)	0.720 (0.672-0.764)	0.689	0.838 (0.795-0.874)	0.000	0.776 (0.730-0.816)	0.105	0.806	0.828 (0.782-0.866)	0.000	0.709 (0.660-0.754)	0.799	0.764
**Fibrosis**	0.590 (0.533-0.646)	0.601 (0.543-0.656)	0.595	0.469 (0.410-0.528)	0.004	0.449 (0.392-0.507)	0.000	0.458	0.705 (0.625-0.775)	0.022	0.346 (0.293-0.403)	0.000	0.464
**Fracture**	0.861 (0.806-0.902)	0.762 (0.703-0.813)	0.808	0.868 (0.794-0.919)	0.848	0.436 (0.373-0.501)	0.000	0.581	0.753 (0.687-0.809)	0.007	0.630 (0.565-0.690)	0.000	0.686
**Mass**	0.605 (0.456-0.736)	0.722 (0.560-0.842)	0.658	0.595 (0.435-0.737)	0.927	0.611 (0.449-0.752)	0.227	0.603	0.719 (0.546-0.844)	0.304	0.639 (0.476-0.775)	0.508	0.676
**Nodule**	0.417 (0.392-0.443)	0.468 (0.441-0.495)	0.441	0.411 (0.380-0.443)	0.789	0.306 (0.281-0.331)	0.000	0.351	0.291 (0.269-0.314)	0.000	0.368 (0.342-0.395)	0.001	0.325
**Pleural thickening**	0.506 (0.429-0.584)	0.612 (0.526-0.692)	0.554	0.379 (0.291-0.475)	0.043	0.302 (0.230-0.386)	0.000	0.336	0.430 (0.340-0.525)	0.222	0.357 (0.279-0.442)	0.000	0.390
**Pneumatosis**	0.500 (0.188-0.812)	0.231 (0.082-0.503)	0.316	0.273 (0.097-0.566)	0.349	0.231 (0.082-0.503)	1.000	0.250	0.300 (0.108-0.603)	0.424	0.231 (0.082-0.503)	1.000	0.261
**Pneumothorax**	0.783 (0.581-0.903)	0.581 (0.408-0.736)	0.667	0.652 (0.449-0.812)	0.326	0.484 (0.320-0.652)	0.774	0.556	0.618 (0.450-0.761)	0.189	0.677 (0.501-0.814)	0.549	0.646
**Postoperative Metal**	0.891 (0.770-0.953)	0.932 (0.818-0.977)	0.911	0.896 (0.778-0.955)	0.943	0.977 (0.882-0.996)	0.625	0.935	0.929 (0.810-0.975)	0.544	0.886 (0.760-0.950)	1.000	0.907
**Venipuncture**	0.923 (0.667-0.986)	0.800 (0.548-0.930)	0.857	0.933 (0.702-0.988)	0.916	0.933 (0.702-0.988)	0.500	0.933	0.867 (0.621-0.963)	0.630	0.867 (0.621-0.963)	1.000	0.867
**Mean**	0.640	0.639	0.632	0.608	-	0.529	-	0.559	0.632	-	0.555	-	0.586

The precision of different abnormality between YOLOX models and radiologist (Junior or Senior) were performed with chi-square test (two-sided).

The recall of different abnormality between YOLOX models and radiologist (Junior or Senior) were performed with related-samples McNemar test (two-sided).

The 95% Wilson confidence interval was applied to both the precision and recall of different abnormality.

IoU-T: intersection over union threshold.

(Source data are provided as a Source Data file.)

Supplementary Table 19 Performance of the other four radiologists on the recombination test dataset with an IoU-T of 0.5.

	Junior 2			Junior 3			Senior 1			Senior 3		
	**Precision**	**Recall**	**F1**	**Precision**	**Recall**	**F1**	**Precision**	**Recall**	**F1**	**Precision**	**Recall**	**F1**
**Atelectasis**	0.615 (0.355-0.823)	0.615 (0.355-0.823)	0.615	0.636 (0.354-0.848)	0.538 (0.291-0.768)	0.583	0.571 (0.326-0.786)	0.615 (0.355-0.823)	0.593	0.636 (0.354-0.848)	0.538 (0.291-0.768)	0.583
**Calcification**	0.723 (0.675-0.766)	0.445 (0.405-0.485)	0.551	0.725 (0.683-0.764)	0.557 (0.517-0.596)	0.630	0.695 (0.652-0.734)	0.570 (0.530-0.609)	0.626	0.671 (0.629-0.711)	0.564 (0.524-0.603)	0.613
**Consolidation**	0.548 (0.503-0.592)	0.603 (0.556-0.648)	0.574	0.559 (0.515-0.603)	0.629 (0.582-0.673)	0.592	0.566 (0.519-0.611)	0.584 (0.537-0.630)	0.575	0.709 (0.658-0.755)	0.551 (0.504-0.598)	0.620
**Effusion**	0.495 (0.424-0.566)	0.386 (0.327-0.449)	0.434	0.437 (0.362-0.515)	0.286 (0.233-0.346)	0.346	0.484 (0.413-0.555)	0.369 (0.311-0.432)	0.419	0.455 (0.375-0.536)	0.270 (0.218-0.329)	0.339
**Emphysema**	0.878 (0.836-0.911)	0.701 (0.652-0.746)	0.780	0.871 (0.827-0.906)	0.676 (0.626-0.722)	0.761	0.881 (0.841-0.912)	0.781 (0.736-0.821)	0.828	0.886 (0.844-0.918)	0.709 (0.660-0.754)	0.788
**Fibrosis**	0.720 (0.638-0.789)	0.336 (0.283-0.393)	0.458	0.493 (0.429-0.558)	0.396 (0.341-0.454)	0.439	0.517 (0.449-0.584)	0.382 (0.327-0.439)	0.439	0.547 (0.486-0.607)	0.491 (0.433-0.549)	0.518
**Fracture**	0.782 (0.702-0.846)	0.427 (0.365-0.492)	0.553	0.821 (0.741-0.880)	0.423 (0.360-0.488)	0.558	0.808 (0.742-0.861)	0.595 (0.530-0.656)	0.685	0.656 (0.589-0.716)	0.612 (0.548-0.673)	0.633
**Mass**	0.739 (0.535-0.875)	0.472 (0.320-0.630)	0.576	0.720 (0.524-0.857)	0.500 (0.345-0.655)	0.590	0.714 (0.529-0.847)	0.556 (0.396-0.705)	0.625	0.895 (0.686-0.971)	0.472 (0.320-0.630)	0.618
**Nodule**	0.516 (0.472-0.559)	0.206 (0.185-0.229)	0.295	0.402 (0.373-0.432)	0.328 (0.302-0.354)	0.361	0.373 (0.343-0.404)	0.282 (0.258-0.307)	0.321	0.366 (0.337-0.396)	0.290 (0.266-0.315)	0.324
**Pleural thickening**	0.667 (0.530-0.780)	0.264 (0.195-0.346)	0.378	0.213 (0.169-0.267)	0.442 (0.359-0.528)	0.288	0.207 (0.159-0.264)	0.364 (0.286-0.450)	0.264	0.233 (0.186-0.288)	0.473 (0.389-0.559)	0.312
**Pneumatosis**	0.667 (0.300-0.903)	0.308 (0.127-0.576)	0.421	0.444 (0.189-0.733)	0.308 (0.127-0.576)	0.364	0.286 (0.082-0.641)	0.154 (0.043-0.422)	0.200	0.333 (0.121-0.646)	0.231 (0.082-0.503)	0.273
**Pneumothorax**	0.556 (0.373-0.724)	0.484 (0.320-0.652)	0.517	0.600 (0.387-0.781)	0.387 (0.237-0.562)	0.471	0.739 (0.535-0.875)	0.548 (0.378-0.708)	0.630	0.625 (0.453-0.771)	0.645 (0.469-0.789)	0.635
**Postoperative Metal**	0.840 (0.715-0.917)	0.955 (0.849-0.987)	0.894	0.857 (0.733-0.929)	0.955 (0.849-0.987)	0.903	0.976 (0.877-0.996)	0.932 (0.818-0.977)	0.953	0.913 (0.797-0.966)	0.955 (0.849-0.987)	0.933
**Venipuncture**	0.786 (0.524-0.924)	0.733 (0.480-0.891)	0.759	0.867 (0.621-0.963)	0.867 (0.621-0.963)	0.867	0.867 (0.621-0.963)	0.867 (0.621-0.963)	0.867	0.857 (0.601-0.960)	0.800 (0.548-0.930)	0.828
**Mean**	0.681	0.495	0.557	0.618	0.521	0.554	0.620	0.543	0.573	0.627	0.543	0.573

The 95% Wilson confidence interval was applied to both the precision and recall of different abnormality. IoU-T: intersection over union threshold.

(Source data are provided as a Source Data file.)

Supplementary Table 20 ANOVA for F1-score achieved by the YOLOX model, junior group and senior group on the recombination test dataset with an IoU-T of 0.5.

	Sum of Squares	df	Mean Square	F	*P*
**Between Groups**	0.060	2	0.030	0.808	0.449
**Within Groups**	3.518	95	0.037		
**Total**	3.578	97			

Comparisons were performed with a two-sided Analysis of Variance (ANOVA).

Df: Degree of freedom. IoU-T: intersection over union threshold.

(Source data are provided as a Source Data file.)

Supplementary Table 21 Multiple comparisons among the YOLOX model, junior group and senior group on the recombination test dataset with an IoU-T of 0.5.

		Mean Difference			95% Confidence Interval	
(I) Group	(J) Group	(I-J)	Std. Error	*P*	Lower Bound	Upper Bound
**Junior group**	**Senior group**	-0.021	0.042	0.624	-0.104	0.063
**Senior group**	**YOLOX**	-0.055	0.059	0.358	-0.173	0.063
**YOLOX**	**Junior group**	0.075	0.059	0.207	-0042	0.193

*. The mean difference is significant at the 0.05 level. Multiple comparisons were performed with a two-sided LSD method.

(Source data are provided as a Source Data file.)

Supplementary Table 22 The number of false positive finding of each abnormality on different test datasets with different IoU-Ts

Test dataset	IoU-T	Atelectasis	Calcification	Consolidation	Effusion	Emphysema	Fibrosis	Fracture	Mass	Nodule	Pleural thickening	Pneumatosis	Pneumothorax	Postoperative metal	Venipuncture	Total	Average FP per CXR
**Held-out (n=6000)**	0.1	13	146	241	275	329	203	99	30	682	79	48	13	14	17	2189	0.365
	0.3	13	195	261	266	329	208	99	32	816	117	43	17	16	16	2428	0.405
	0.5	6	301	254	426	329	244	117	27	1245	67	72	23	28	27	3166	0.528
**External dataset A (n=2978)**	0.1	3	50	62	92	58	45	20	5	284	29	6	0	13	1	668	0.224
	0.3	3	62	67	96	58	52	20	5	308	30	9	1	13	1	725	0.243
	0.5	3	90	81	146	58	62	38	3	423	19	8	5	13	1	950	0.319
**External dataset B (n=2633)**	0.1	6	53	112	70	67	79	23	11	429	28	7	4	2	0	891	0.338
	0.3	1	60	124	79	67	39	16	11	503	29	8	6	2	0	945	0.359
	0.5	1	89	160	118	67	52	17	11	646	45	5	7	2	0	1220	0.463
**External dataset C (n=2651)**	0.1	3	142	285	138	324	107	63	19	870	46	6	7	1	3	2014	0.760
	0.3	4	159	307	145	324	95	65	24	919	65	6	7	1	3	2124	0.801
	0.5	4	208	354	209	324	113	69	26	1142	56	6	8	1	0	2520	0.951
**External dataset D (n=2683)**	0.1	4	118	120	96	299	35	46	19	742	24	13	6	0	1	1523	0.568
	0.3	4	91	128	100	299	38	47	19	783	26	16	6	0	1	1558	0.581
	0.5	5	119	141	142	299	52	54	19	969	33	24	8	0	1	1866	0.695
**Recombination test dataset (n=2800)**	0.1	11	73	168	113	134	97	26	13	597	60	2	4	5	1	1304	0.466
	0.3	12	87	193	124	134	109	26	14	641	61	3	4	5	1	1414	0.505
	0.5	9	111	217	185	134	118	28	17	835	77	3	5	5	1	1745	0.623
**Prospective dataset (n=1540)**	0.1	1	84	50	88	82	24	13	4	136	15	17	5	14	2	535	0.347
	0.3	1	62	50	101	82	24	13	4	90	15	15	6	15	2	480	0.311
	0.5	1	77	36	130	82	30	14	4	91	18	28	11	15	3	540	0.350

FP: False positive. IoU-T: intersection over union threshold.

(Source data are provided as a Source Data file.)

Supplementary Table 23 The general information about each dataset used for the development and validation of the CTR calculation algorithm.

	Training and tuning dataset	Test dataset	External dataset A	External dataset B	External dataset C	External dataset D	Prospective dataset
**Collected CXR**	10000	2000	500	500	500	500	300
**Included CXR**	9574	1950	498	495	484	492	293
**Patients**	9063	1944	498	491	477	491	292
**Age (mean ±sd)**	55.0±15.9	57.9±15.0	47.0±17.4	50.5±16.7	59.3±15.0	53.9±16.9	55.7±15.5
**Gender (males, %)**	5473(57.165%)	1116(57.231%)	218(43.775%)	247(49.899%)	273(56.405%)	270(54.878%)	140(47.728%)

CTR: cardiothoracic ratio.

(Source data are provided as a Source Data file.)

Supplementary Table 24 Manufacturer and device information concerning the CXRs used for the development and validation of the CTR calculation algorithm.

Manufacturer information	Device information	Training and tuning dataset	Test dataset	External dataset A	External dataset B	External dataset C	External dataset D	Prospective dataset
Shimadzu	RADspeed Pro 50	0	0	1	493	0	0	0
Carestream Health	DRX Evolution	680	244	0	0	180	0	0
United-imaging Health	uDR 770i	0	0	0	0	0	0	128
KODAK	DirectView DR7500	1310	187	0	0	0	0	56
	DirectView DR3500	0	0	356	0	0	0	0
	DirectView DR3000	0	0	0	0	304	0	0
SIEMENS	AXIOM Aristos VX Plus	7584	1519	0	0	0	0	109
	Multix Fusion Max	0	0	141	0	0	0	0
DDIT	WV3000T	0	0	0	2	0	0	0
General Medical Merate S.p.A.	CALPYSO	0	0	0	0	0	492	0
Total		9574	1950	498	495	484	492	293

CTR: cardiothoracic ratio.

(Source data are provided as a Source Data file.)

Supplementary Table 25 Annotation principles for each abnormality in CXRs.

Abnormality	Implication of abnormality	Annotation principle
**Atelectasis**	A complete or partial collapse of the entire lung or an area (lobe) of the lung.	The minimum external bounding box containing the pulmonary atelectasis.
**Calcification**	Pulmonary calcification, aortic arch calcification, pleural calcification, or lymph node calcification. Excludes calcification of the costal cartilage.	If calcification is scattered, it needs to be separately labelled with the minimum external bounding box. Pleural calcification and pleural thickening often overlap and need to be independently labelled with the minimum external bounding box.
**Consolidation**	Exudation, oedema, or infection that can lead to consolidation.	The minimum external bounding box that contains whole consolidation region.
**Effusion**	Includes encapsulated pleural effusion, interlobar fissure effusion and free pleural effusion.	For free pleural effusion, the upper boundary is the upper arc margin of the effusion, the lateral boundary is the intersection point of the effusion and the thorax, the lower margin is determined according to the position of the contralateral diaphragm, and the medial margin is determined according to the junction point of the effusion and the diaphragm. Other types of effusion are annotated according to the minimum external bounding box.
**Emphysema**	Enhanced translucency of the lung field.	The minimum external bounding box including the upper, lower, left, and right boundaries of both lungs.
**Fibrosis**	Reticular, threadlike, and cable-like shadows on CXRs when a lung is damaged and scarred.	According to the shape of the fibrosis, the minimum external bounding box is annotated.
**Fracture**	Bone discontinuities including old and fresh fractures.	The bounding box should contain the fracture line and two broken ends of the fracture.
**Mass**	A round or round-like opacity $>$ 3 cm in diameter.	Determined according to the edge of the mass annotated with the minimum external bounding box.
**Nodule**	A round or round-like opacity $\leq$ 3 cm in diameter.	Determined according to the edge of the nodule annotated with the minimum external bounding box. For nodules smaller than 1 cm, the minimum external bounding box can be appropriately enlarged.
**Pleural thickening**	Any form of thickening by the scar tissue on the parietal pleura or pulmonary pleura.	According to the shape of pleural thickening, the minimum external bounding box is annotated.
**Pneumatosis**	Subcutaneous soft tissue pneumatosis, pneumomediastinum, pneumopericardium, subphrenic free gas, pulmonary bullae, or gas in a cavity.	According to the shape of the pneumatosis, the minimum external bounding box is annotated.
**Pneumothorax**	The presence of gas (air) in the pleural cavity (the space around the lungs).	According to the compression edge of the lung tissue, the minimum external bounding box of the pneumothorax annotation is determined.
**Postoperative Metal**	Internal fixation of fractures, metal cardiac prosthetic valves, metal arterial stents, metal electrode tips for pacemakers, or bone cement. Metal sutures to the sternum are excluded.	For a pacemaker electrode, the associated bounding box only contains its tip. Because the pacemaker tip electrode is small, the minimum external rectangular box can be appropriately enlarged. Other types of postoperative metal are annotated according to their shapes with the minimum external bounding boxes.
**Venipuncture**	Peripherally inserted central catheter (PICC) or deep vein catheterization.	The tip of the PICC or deep vein catheterization is annotated in the centre of a bounding box with a size of approximately about 1 cm×1 cm.

Supplementary Table 26 The number of CXRs with multiple abnormalities in the CXR-AL14 dataset

	Normal	single abnormality	2 abnormalities	3 abnormalities	4 abnormalities	5 abnormalities	6 abnormalities	7 abnormalities	8 abnormalities	Total
**CXRs**	63084	47016	28683	16369	7546	2537	613	118	22	165988

Supplementary Table 27 The number of CXRs per iteration during the construction of the CXR-AL14 dataset

	Preliminary model	Iteration 1	Iteration2	Iteration 3	Iteration 4	Iteration 5	Iteration 6	Iteration 7	Total
**CXRs**	8000	24684	23761	24118	22186	23574	21694	17971	165988

Supplementary Table 28 Number of CXRs and each abnormality annotated by each expert.

	Expert group	Expert 1	Expert 2	Expert 3	Expert 4	Expert 5	Expert 6	Total
**CXRs**	8000	26329	26329	26332	26332	26332	26334	165988
**Atelectasis**	17	61	61	57	52	36	74	358
**Calcification**	1640	6278	5471	5254	5441	5015	4255	33354
**Consolidation**	1002	3533	3281	3135	2921	2797	4597	21266
**Effusion**	2015	6847	6488	6854	6421	6519	8967	44111
**Emphysema**	1159	3779	3966	4057	4027	3718	2884	23590
**Fibrosis**	750	2835	2486	2452	2490	2301	3373	16687
**Fracture**	575	2328	1927	1907	1793	1564	2538	12632
**Mass**	132	469	436	416	394	441	710	2998
**Nodule**	2170	8724	6518	6620	7771	7131	7043	45977
**Pleural thickening**	336	1685	1084	1218	962	872	1236	7393
**Pneumatosis**	259	461	739	839	1068	1125	2462	6953
**Pneumothorax**	313	715	941	910	1002	1094	2089	7064
**Postoperative Metal**	866	3679	2629	2644	2884	2970	4257	19929
**Venipuncture**	482	894	1230	1472	1784	2004	3666	11532
**Total**	11716	42288	37257	37835	39010	37587	48151	253844
