# Supplementary material for: A deep-learning-based framework for identifying and localizing multiple abnormalities and assessing cardiomegaly in chest X-ray
Source: Nat Commun. 2024 Feb 14;15:1347. doi: 10.1038/s41467-024-45599-z (PMC10867134; doi:10.1038/s41467-024-45599-z)
Supplement: Supplementary file 3 — Reporting Summary [file 41467_2024_45599_MOESM3_ESM.pdf]

# nature portfolio

Corresponding author(s): Yongjian Nian and Dong Zhang

Last updated by author(s): Jan 28, 2024

# Reporting Summary

Nature Portfolio wishes to improve the reproducibility of the work that we publish. This form provides structure for consistency and transparency in reporting. For further information on Nature Portfolio policies, see our Editorial Policies and the Editorial Policy Checklist.

## Statistics

For all statistical analyses, confirm that the following items are present in the figure legend, table legend, main text, or Methods section.

| n/a | Confirmed | |
|---|---|---|
| ☐ | ☒ | The exact sample size ($n$) for each experimental group/condition, given as a discrete number and unit of measurement |
| ☐ | ☒ | A statement on whether measurements were taken from distinct samples or whether the same sample was measured repeatedly |
| ☐ | ☒ | The statistical test(s) used AND whether they are one- or two-sided<br>*Only common tests should be described solely by name; describe more complex techniques in the Methods section.* |
| ☒ | ☐ | A description of all covariates tested |
| ☐ | ☒ | A description of any assumptions or corrections, such as tests of normality and adjustment for multiple comparisons |
| ☐ | ☒ | A full description of the statistical parameters including central tendency (e.g. means) or other basic estimates (e.g. regression coefficient) AND variation (e.g. standard deviation) or associated estimates of uncertainty (e.g. confidence intervals) |
| ☐ | ☒ | For null hypothesis testing, the test statistic (e.g. $F$, $t$, $r$) with confidence intervals, effect sizes, degrees of freedom and $P$ value noted<br>*Give P values as exact values whenever suitable.* |
| ☒ | ☐ | For Bayesian analysis, information on the choice of priors and Markov chain Monte Carlo settings |
| ☐ | ☒ | For hierarchical and complex designs, identification of the appropriate level for tests and full reporting of outcomes |
| ☒ | ☐ | Estimates of effect sizes (e.g. Cohen's $d$, Pearson's $r$), indicating how they were calculated |

*Our web collection on statistics for biologists contains articles on many of the points above.*

## Software and code

Policy information about availability of computer code

| | |
|---|---|
| Data collection | All the CXRs of CXR-AL14 dataset and held-out test dataset were collected from the Second Affiliated Hospital of Army Medical University, and were taken by the following three devices: DR Aristotle VX Plus (Siemens, Germany), Evolution DR (Carestream Health, Canada) and DirectView (Kodak, USA). The CXRs of multicentre test datasets were randomly collected from four hospitals. We randomly selected 700 CXRs from each centre, and in a total of 2800 CXRs to construct the recombination test dataset. After the patients signed the informed consent form, their posteroanterior CXRs were collected from the Second Affiliated Hospital of Army Medical University as the propective test dataset . Both the category and localization of each abnormality in above CXRs were annotated by the LabelImg tool (v1.8.0, https://pypi.org/project/labelImg/1.8.0/). |
| Data analysis | The program codes of the YOLOX model and the CTR calculation algorithm in this study are publicly available, which can be downloaded at https://github.com/CXR-AL14/CXR-Code. DOI link: https://doi.org/10.5281/zenodo.8120660.<br>The general informations of CXRs in the CXR-AL14 dataset were conducted using numpy package (version 1.19.3) in Python (version 3.7.9 ). The McNemar test and chi-square test were carried out using statsmodels (version 0.13.2) and scipy (version 1.5.4) packages in Python (version 3.7.9). ANOVA and the LSD method were conducted using SPSS Statistics (Version 22.0.0, IBM SPSS Statistics, Armonk, New York). |

For manuscripts utilizing custom algorithms or software that are central to the research but not yet described in published literature, software must be made available to editors and reviewers. We strongly encourage code deposition in a community repository (e.g. GitHub). See the Nature Portfolio guidelines for submitting code & software for further information.

## Data

Policy information about availability of data

All manuscripts must include a data availability statement. This statement should provide the following information, where applicable:

- Accession codes, unique identifiers, or web links for publicly available datasets
- A description of any restrictions on data availability
- For clinical datasets or third party data, please ensure that the statement adheres to our policy

All data supporting the findings described in this manuscript are available in the article and in the Supplementary Information. According to relevant national regulations, there are certain restrictions on the number of medical images for publicly available. Therefore, the CXR-AL14 dataset is partially available for public use (nearly 100,000 CXRs), interested researchers can contact the corresponding author Dong Zhang (hszhangd@tmmu.edu.cn.) or visit this website [cxr-al14.top] to request access. In addition, the total CXR-AL14 dataset is available for online use by requested on the website [https://www.ncmi.cn//phda/dataDetails.do?id=CSTR:17970.11.A0048.202312.605.V1.0]. It should be noted that the CXR-AL14 dataset will only be available for academic research, and not for other purposes. Interested researchers need to register their personal and institutional information on above websites and send data access requests to the web administrator. The web administrator and corresponding author will review the requests for consideration and respond within two weeks. Once approved, the dataset can be used by the interested researchers. Note that interested researchers who have utilized the CXR-AL14 dataset for research must cite this article.

The multicentre test datasets are not available for public use. If the interested researchers want to achieve the multicentre test datasets for non-commercial use, they can request for the corresponding author Dong Zhang (hszhangd@tmmu.edu.cn.). Corresponding author will review their requests and ask for consent from each centre, requestors will receive a response within two weeks.

Source data are provided with this paper.

The program codes of the YOLOX model and the CTR calculation algorithm in this study are publicly available, which can be downloaded at https://github.com/CXR-AL14/CXR-Code. DOI link: https://doi.org/10.5281/zenodo.8120660.

## Human research participants

Policy information about studies involving human research participants and Sex and Gender in Research.

| | |
|---|---|
| Reporting on sex and gender | In this study, sex and gender were not considered separately. Both CXRs form male and CXRs from female were incorporated into the model training without discrimination. In the process of data statistics, we counted gender data in the training dataset, testing dataset and each test dataset. The framework presented in this paper applies equally to male and female. |
| Population characteristics | We consecutively collected 315,072 original CXRs from 159,996 patients, after exclusion, the CXR-AL14 dataset was created containing 165,988 CXRs from 144,968 patients. The mean age of the patients was 53.44±15.20 years. 92,954 CXRs of patients (56.000%) were male, and 73,034 CXRs of patients (44.000%) were female. |
| Recruitment | We consecutively collected 315,072 original CXRs from 159,996 patients at the Department of Radiology, the Second Affiliated Hospital of Army Medical University, between August 2011 and December 2021. Multicentre test datasets were randomly collected from four hospitals, and the recombination test dataset was randomly selected from each centre. The CXRs of propective test dataset were collected after the patients signed the informed consent form. This method had nearly no self-selection bias or other bias. |
| Ethics oversight | This study was approved by the Medical Ethics Committee of the Second Affiliated Hospital of Army Medical University (no. 2021-159-01, no. 2022-193-01 and no. 2023-123-01). All methods were implemented in accordance with the approved regulations and the Declaration of Helsinki. The CXR-AL14 dataset and held-out test dataset were collected retrospectively with a waiver granted for the requirement of informed consent (no. 2021-159-01). The multicentre validation in this study was approved by the Medical Ethics Committee of the principal investigator's hospital. The multicentre hospital retrospectively collected CXR data according to the approved experimental procedures (no. 2022-193-01), and informed consent was waived for this retrospective analysis. Moreover, the CXRs of all the retrospective datasets including the CXR-AL14 dataset, held-out, and multicentre test datasets were de-identified to remove any patient-related information before collection. None of the authors participated in the data de-identification process. The prospective test dataset was collected prospectively in accordance with procedures approved by the hospital Ethics Committee (no. 2023-123-01) and written informed consent was obtained from each participant. The CXRs of prospective test datasets were also de-identified before transfer to study investigators. |

Note that full information on the approval of the study protocol must also be provided in the manuscript.

# Field-specific reporting

Please select the one below that is the best fit for your research. If you are not sure, read the appropriate sections before making your selection.

[x] Life sciences [ ] Behavioural & social sciences [ ] Ecological, evolutionary & environmental sciences

For a reference copy of the document with all sections, see nature.com/documents/nr-reporting-summary-flat.pdf

# Life sciences study design

All studies must disclose on these points even when the disclosure is negative.

| | |
|---|---|
| Sample size | The CXR-AL14 dataset contains 165,988 CXRs (102,904 abnormal CXRs and 63,084 “No finding” CXRs) with 253,844 GT bounding boxes for 14 chest common abnormalities. We employed an approach named human-in-the-loop in which humans and models work in tandem to help expert group annotating CXRs of CXR-AL14 dataset. During this processed, as the number of iterations increased, the number of annotated CXRs also increased, and the performance of the updated model gradually improved. After seven iterations, the performance improvement of the updated model tends to be stable, so the sample size is not expanded and the construction of CXR-AL14 was completed. The updated model after the seventh iteration was just the YOLOX model in proposed framework, which achieved a good performance. |
| Data exclusions | Lateral or anteroposterior CXRs, CXRs from patients under the age of eighteen, and duplicate CXRs were excluded from the study. |
| Replication | To assess the reproducibility of the YOLOX model, 5-fold cross-validation was further performed on the CXR-AL14 dataset, and the five trained models were further tested on the held-out test dataset. The results demonstrated that the repeatability of our study is good. |
| Randomization | First, 6,000 CXRs were randomly selected from filtered CXRs as a held-out test dataset. Second, during the training of the YOLOX model, we randomly divided all CXRs from CXR-AL14 into training and tuning datasets at a ratio of 9:1. Third, Multicentre validation datasets were randomly collected from four hospitals, and 700 CXRs from each multicentre were randomly selected to construct the recombination test dataset (a total of 2800 CXRs) for a further performance comparison between the YOLOX model and radiologists. Finally, the training/testing datasets were randomly selected from included CXRs to deveop and validate an automatic CTR calculation algorithm. |
| Blinding | The investigators were blinded to the group allocation during data collection and/or analysis. |

# Reporting for specific materials, systems and methods

We require information from authors about some types of materials, experimental systems and methods used in many studies. Here, indicate whether each material, system or method listed is relevant to your study. If you are not sure if a list item applies to your research, read the appropriate section before selecting a response.

## Materials & experimental systems

| n/a | Involved in the study | |
|---|---|---|
| ☒ | ☐ | Antibodies |
| ☒ | ☐ | Eukaryotic cell lines |
| ☒ | ☐ | Palaeontology and archaeology |
| ☒ | ☐ | Animals and other organisms |
| ☒ | ☐ | Clinical data |
| ☒ | ☐ | Dual use research of concern |

## Methods

| n/a | Involved in the study | |
|---|---|---|
| ☒ | ☐ | ChIP-seq |
| ☒ | ☐ | Flow cytometry |
| ☒ | ☐ | MRI-based neuroimaging |
